# Nuclear accumulation of PANK4 in hippocampal astrocytes aggravates cuproptosis in association with mild cognitive impairment in aged mice

**DOI:** 10.3389/fnagi.2026.1816702

**Published:** 2026-05-13

**Authors:** Bo Wang, Jing Zhang, Chang-Hong Li, Xiao Huang, Ruo-Bing Gao, Yan Peng, Qing Xie, Ya-Lei Ning, Yan Zhao, Nan Yang, Xing Chen, Yang-Li Xie, Yuan-Guo Zhou, Sen Lin, Lin Chen, Ping Li

**Affiliations:** 1 The Molecular Biology Center, State Key Laboratory of Trauma and Chemical Poisoning, Department of Army Occupational Disease, Daping Hospital, Army Medical University (Third Military Medical University), Chongqing, China; 2 Laboratory for Prevention and Rehabilitation of Training Injuries, State Key Laboratory of Trauma and Chemical Poisoning, Center of Bone Metabolism and Repair, Trauma Center, Daping Hospital, Research Institute of Surgery, Army Medical University (Third Military Medical University), Chongqing, China; 3 Department of Anatomy, Anhui Medical University, Hefei, Anhui, China; 4 Department of Anesthesiology, Sichuan Academy of Medical Sciences and Sichuan Provincial People’s Hospital, University of Electronic Science and Technology of China, Chengdu, China; 5 Laboratory of stem cell and Tissue Engineering, Chongqing Medical University, Chongqing, China; 6 Department of Neurology, Xinqiao Hospital, The Second Affiliated Hospital, Army Medical University (Third Military Medical University), Chongqing, China

**Keywords:** aged mouse, cuproptosis, mild cognitive impairment, nuclear accumulation, PANK4

## Abstract

**Background:**

Mild cognitive impairment (MCI), a condition that falls somewhere between normal aging and severe cognitive dysfunction (e.g., Alzheimer’s disease), is a common manifestation of the neurocognitive function decline that seniors encounter as they age. The fundamental processes causing its beginning are still not well understood yet.

**Methods:**

We employed aged (18-month-old) male C57BL/6J mice, including astrocyte-specific Pantothenate kinases 4 (PANK4) conditional knockout (Pank4^*f/f*^;Gfap-Cre, Pank4-CKO) mice. Cognitive function was assessed using the Barnes maze, Y-maze (spatial novelty preference, spontaneous alternation), novel object recognition (NOR) test, and open field test (OFT). Hippocampal PANK4 localization was analyzed via immunofluorescence (IF) and subcellular fractionation/western blotting (WB). Cuproptosis markers (FDX1, LIAS, DLAT), copper transporters (ATP7A, ATP7B, SLC31A1), and copper content (ICP-MS) were quantified in hippocampal tissue. *In vitro* studies used LPS-stimulated primary astrocytes for RNA-seq and qPCR validation.

**Results:**

Aged wild-type (18M+WT) mice exhibited specific deficits in Barnes maze retention and reversal learning, indicative of mild cognitive impairment, while Pank4-CKO mice showed significant rescue. We discovered a novel age-dependent nuclear accumulation of PANK4 in hippocampal cells, which was absent in Pank4-CKO mice. Aged hippocampi displayed upregulated pro-cuproptotic factors (FDX1, LIAS) and reduced DLAT, alongside decreased expression of the copper exporter ATP7A, ATP7B, SLC31A1 and increased copper accumulation. Astrocyte-specific Pank4 knockout reversed these changes: it suppressed FDX1/LIAS upregulation, restored ATP7B expression and DLAT levels, and normalized hippocampal copper content. *In vitro*, LPS-induced neuroinflammation triggered PANK4 nuclear translocation and selectively downregulated Atp7b expression in astrocytes. Small interfering RNA (siRNA)-mediated knockdown of Pank4 significantly upregulated Atp7b expression.

**Conclusion:**

This study identifies a novel pathological mechanism in age-related MCI: the nuclear accumulation of PANK4 in hippocampal exacerbates cuproptosis susceptibility by specifically impairing ATP7B-dependent copper efflux, leading to copper overload. Astrocyte-specific PANK4 ablation mitigates these effects, highlighting PANK4 as a potential therapeutic target for preventing or treating age-associated cognitive decline.

## Introduction

Aging represents a critical global health challenge, characterized by a multifaceted biological process entailing progressive physiological decline across multiple organ systems.^1^ The brain exhibits heightened vulnerability to these age-related alterations, often manifesting as impairments in core cognitive domains, including progressive memory deficits and diminished learning capacity (Tarailis et al., 2025; Umoh, 2025; Wu and Chen, 2026). Mild cognitive impairment (MCI), a well-recognized early clinical manifestation, is defined by objectively measurable declines in memory or other cognitive functions that exceed normative age-related changes, yet do not significantly impair independent activities of daily living (Golomb et al., 2004; Liu et al., 2025; Overton et al., 2019; Petersen et al., 1995). MCI is thus established as an intermediate clinical state (or the prodromal stage) between normative cognitive aging and dementia (most common form is Alzheimer’s disease, AD) (Janoutová et al., 2015; Lin et al., 2020). AD’s pathophysiology is complex and heterogeneous, involving mechanisms such as amyloid-beta (Aβ) plaque deposition, neurofibrillary tangle formation, synaptic dysfunction, neuronal loss, neuroinflammation, and cerebrovascular pathology (Li et al., 2024; Patel et al., 2025). Consequently, early identification and intervention in MCI are critical for slowing AD’s progression. Notably, to date, no pharmacological agents have been formally approved for the treatment of MCI. Identifying neuroprotective factors that mitigate age-associated neurocognitive decline and preserve cognitive function comparable to younger cohorts is of paramount importance.

Pantothenate kinases 1, 2, and 3 (PANK1-3) are crucial regulatory enzymes within coenzyme A (CoA) biosynthesis (Barritt et al., 2024). They mediate phosphorylation of vitamin pantothenate, yielding 4′-phosphopantothenate—the initial step toward CoA production. Significantly, recessive congenital PANK2 mutations cause a specific neurodegeneration with brain iron accumulation subtype, termed PANK-associated neurodegeneration (PKAN) (Li et al., 2022; Zizioli et al., 2016). In contrast, the fourth family member, PANK4, exhibits no enzymatic activity. This functional absence stems from a missing catalytic glutamate residue essential for catalysis (Hörtnagel et al., 2003; Yao et al., 2019). Interestingly, inhibiting PANK4 phosphorylation relieves its suppressive effect on CoA synthesis (Dibble et al., 2022) and increasing PANK4 levels enhances glucose uptake in skeletal muscle (Miranda-Cervantes et al., 2025), representing a distinct regulatory feature.

While PANK4’s cellular and organismal functions are inadequately explored, Mei et al. found PANK4 as one of ten proteins exhibiting significant sex-by-trait interactions in AD (Mei et al., 2025). Elucidating the specific roles of PANK4 in AD pathogenesis is therefore warranted, as it holds promise as a potential sex-specific biomarker for future AD diagnostics and research. Pank4 knockout (KO) adult mice exhibited greater resistance against lens fibrosis, a known cataractogenic process (common in the elderly) compared to wild-type (WT) littermates (Li et al., 2023b). But lens acetyl-CoA concentrations showed no significant difference between KO and WT groups (Li et al., 2023b). Investigations using lens epithelial cells revealed that PANK4 deficiency promotes apoptotic cell death (Sun et al., 2019) and elevates glycolytic flux, mediated by altered pyruvate kinase isoform 2 activity (Li et al., 2023b). Interestingly, functional assays indicate PANK4 also modulates pancreatic β-cell apoptosis (Xiang et al., 2007). Consequently, the precise physiological roles and underlying molecular mechanisms of PANK4 thus remain incompletely understood.

Emerging clinical evidence indicates significant dysregulation of copper homeostasis in MCI. Copper (Cu), a redox-active metal, participates in numerous metabolic processes within the brain under physiological conditions (Lei et al., 2021). In human tissues, it may be found as either non-protein-bound Cu (free Cu) or protein-bound Cu, and it acts in the free form (Singh et al., 2012). According to Matos et al., creating a premature aging model (CuSO4-SIPS) with copper sulfate (CuSO4) shows how Cu contributes to age-related functional loss and the progression of age-related illnesses (Matos et al., 2015, 2017). Cohort studies demonstrate elevated circulating copper concentrations in MCI patients relative to cognitively intact controls, while iron levels remain unaltered (Qing et al., 2025). Squitti et al. found a significantly increased nonbound ceruloplasmin copper – a potential diagnostic indicator for identifying MCI cases at elevated risk of progressive cognitive decline (from MCI to AD) (Squitti et al., 2014). Serum copper levels demonstrate an inverse correlation with working memory and executive function in healthy individuals (Mazhari et al., 2020). Concentrations are significantly elevated in elderly subjects with cognitive impairment (Kazemi et al., 2022), suggesting copper dyshomeostasis constitutes a modifiable risk factor in prodromal neurodegeneration.

Copper overload induces cuproptosis, a copper-dependent, non-apoptotic form of regulated cell death driven by lipoylated mitochondrial proteins, which presents novel therapeutic avenues (Tsvetkov et al., 2022). Emerging evidence implicates copper-mediated cuproptosis in the pathogenesis of cognitive dysfunction (Jia et al., 2025; Mo et al., 2025; Zheng M. et al., 2025). For example, cuproptosis has been mechanistically linked to lead (Pb)-induced cognitive impairment, with dihydrolipoamide S-acetyltransferase (DLAT) identified as a critical regulator in this process (Zhao et al., 2025). Furthermore, cuproptosis may contribute to the neuroinflammatory environment characteristic of the AD brain (Mangalmurti and Lukens, 2022). Supporting this, Wang et al. demonstrated in a study that polypyrimidine tract-binding protein 1 (PTBP1) promotes cuproptosis in AD models via the solute carrier family 31 member 1 (SLC31A1) pathway (Wang J. et al., 2025). Nevertheless, the current literature predominantly reports correlative associations between disorders of copper metabolism and AD, lacking direct experimental validation of a causal link between cuproptosis and AD pathogenesis (Wang et al., 2023), and even less so with MCI.

Astrocytes serve critical functions within neural networks governing emotional processing, cognitive development, and memory formation (Feng et al., 2023). Their indispensable roles encompass cellular copper homeostasis, neuronal metabolic support, and modulation of synaptic transmission alongside plasticity regulation (Bhattacharjee et al., 2020). Cuproptosis drives neuropathology by inducing astrocyte dysfunction: (1) It exacerbates experimental cerebral malaria via astrocyte reactivity, suggesting copper modulation as a therapeutic strategy (Hou et al., 2025); (2) It directly bridges copper dysregulation to temporal lobe epilepsy pathology by activating NF-κB-mediated neuroinflammation and neuronal-astrocytic impairment (Li W. et al., 2025); (3) Y2O3 nanoparticles induce cognitive deficits in rats by triggering astrocyte cuproptosis through suppressed TRIM24/DTNBP1/ATP7A copper efflux (Chen et al., 2024). Conversely, astrocyte-derived MT2A upregulation protects against Parkinson’s disease by reducing intracellular Cu^2+^ and dopaminergic neuronal cuproptosis (Cheng et al., 2025). Chen et al. findings may nominate cuproptosis-related lncRNAs (CRLs) and cuproptosis-related genes (CRGs) in astrocyte as prognostic biomarkers and therapeutic targets for glioblastoma (Chen et al., 2025b). Collectively, these findings underscore a close relationship between astrocyte function and cuproptosis. To directly investigate the interplay between cuproptosis and PANK4 in the context of aging-related cognitive decline, we utilized aged (18-month-old) mice and primary astrocyte cultures to assess their impact on aging-associated mild cognitive impairment.

## Materials and methods

### Animals

Male C57BL/6J wild-type mice aged 6–8 or 75 weeks originated from Daping Hospital’s Animal Experimental Center. During CNS development, GFAP expression enables the definitive identification and distinction of astrocytes from other glial cell populations (Stiene-Martin et al., 1991). Owing to its specificity, GFAP was employed as a standard astrocytic marker across our experimental paradigms. Age-matched transgenic males (astrocyte-specific Pank4 knockout, Pank4^*f/f*^;Gfap-Cre) were procured from Cyagen Biosciences. Briefly, Pank4^*f/f*^ animals (gifted by Prof. Sen Lin, Second Affiliated Hospital, Army Medical University) underwent mating with Gfap-Cre mice to produce Pank4^*f/f*^;Gfap-Cre offspring (Li et al., 2023b). Pre-experimental acclimatization spanned 14 days under controlled housing: 20 ± 1°C ambient temperature, 52 ± 2% relative humidity, 12-h light/dark cycles, plus unrestricted access to food/water. Subjects were subsequently categorized into three cohorts (*n* = 11–12/group): Young wild-type (2-month-old, 2M+WT), aged wild-type (18-month-old, 18M+WT), and PANK4 conditional knockout elderly mice (18-month Pank4^*f/f*^;Gfap-Cre, 18M+ Pank4-CKO). All protocols received institutional animal ethics board approval (Army Medical University, AMUWEC20237011), followed ARRIVE reporting guidelines, and aligned with NIH standards for laboratory animal care/utilization.

### Behavioral tests

All behavioral tests were performed during the dark phase (06:30 p.m.–10:00 p.m.).

### Y-maze: spatial novelty preference

The Y-maze apparatus comprises three radially arranged compartments: a designated start compartment (continuously accessible), an alternative accessible compartment (permanently available), and a novelty compartment (barricaded during initial exposure but accessible subsequently). All compartments featured 15-cm high perimeter barriers. Arm angular separation measured 120°. Inter-trial intervals spanned 2 h. Initial habituation sessions permitted 5-min exploration exclusively within start/second compartments while restricting novelty compartment access. Following the inter-trial interval, assessment trials positioned rodents within the start compartment for 5-min unrestricted exploration across all compartments. Overhead digital videography captured movement trajectories, with behavioral quantification via Noldus Ethovision XT 12.1 (Noldus Information Technology, Netherlands).

### Y-maze: spontaneous alternation test

Apparatus specifications mirrored prior Y-maze configurations. Subjects were positioned within the maze’s central hub permitting unrestricted exploration for 5 min. Arm transition events underwent automated quantification via Noldus Ethovision XT 12.1 (Noldus Information Technology, Netherlands). Alternations represented consecutive entries into three distinct compartments. Spatial working memory assessment employed alternation accuracy computed as: [Alternation count ÷ (Total arm entries − 2)] × 100. Accuracy exceeding 50% indicated intact working memory function. Inter-trial sanitation utilized 70% ethanol to eliminate residual olfactory cues.

### Open field test (OFT)

The open-field apparatus (50 × 50 × 45 cm) featured a white PVC-walled arena equipped with infrared photobeam arrays. Mice underwent tail-transfer from home cages to the arena’s central zone. Testing commenced upon locomotion onset triggering beam interruptions, spanning 5 min. Data acquisition utilized Noldus Ethovision XT 12.1 (Noldus Information Technology, Netherlands). The central quadrant (25 × 25 cm) represented the designated analysis region. The total path length quantified spontaneous locomotion, while central zone occupancy duration assessed novelty-induced anxiety per standard paradigm conventions as previously described (Zhang et al., 2024).

### Novel object recognition (NOR) test

NOR assessment executed established methodologies evaluating object recognition memory (Tan et al., 2021). Testing occurred within a 50 × 50 × 45 cm open-field apparatus partitioned into quadrants. The paradigm comprised sequential phases: habituation training and memory retention testing. Pre-training acclimatization allowed 5-min arena exploration without objects. During familiarization trials, two identical non-olfactory fixed stimuli (yellow 5-cmł cubes) were introduced. Subjects explored both objects for 5 min. Retention sessions substituted one familiar stimulus with a novel object (red triangular pyramid), permitting 5-min free investigation. Exploration duration per object underwent videographic recording. Object-directed behavior was operationally defined as nasal/forepaw contact or sniffing ≤ 2 cm proximity (Noldus Ethovision XT 12.1, Netherlands). Recognition memory quantification employed the preferential index: [Novel object exploration duration ÷ total exploration time] × 100%. Alternatively, [Novel object investigation frequency ÷ total investigation events] × 100%. Inter-test sanitation utilized 75% ethanol to neutralize residual odor cues.

### Barnes Maze test

Barnes Maze testing was performed as our previously described (Wang et al., 2020). The equipment features a 122-cm circular platform containing twenty uniformly spaced 5-cm apertures along its perimeter. Rodents escape aversive LED illumination by locating a darkened compartment (designated goal chamber) positioned underneath one aperture. During acquisition trials, subjects were initially confined within a non-transparent cylindrical enclosure centrally positioned for 30 s to induce spatial disorientation. Following cylinder removal, mice freely navigated the apparatus until discovering and accessing the target box. Animals failing to locate the goal chamber within 4 min received manual guidance into it, remaining there ≥ 30 s. Preceding each trial, 75% ethanol wiped all apertures and platform surfaces preventing olfactory cues. Daily training comprised three sessions spaced 15 min apart. Individual trial data underwent averaging. Acquisition spanned four consecutive days. Subsequently, animals experienced two maze-free rest days. On day 7, retention testing employed three trials matching original goal box placement. Reversal trials commenced Day 8: the escape box relocated to a novel quadrant. Mirroring prior procedures, reversal testing involved thrice-daily sessions across days 8–9. Escape latency—interval from cylinder removal to goal box entry—was quantified using Noldus Ethovision XT 12.1 (Noldus Information Technology, Wageningen, The Netherlands).

### Immunofluorescence (IF)

Upon concluding behavioral assessments, three animals per cohort received anesthesia via 4% chloral hydrate. Transcardial perfusion ensued using 0.9% saline succeeded by chilled 4% paraformaldehyde. Excised brains underwent immersion fixation in identical paraformaldehyde (24 h), then dehydration in 30% sucrose solution (2–3 days). Subsequently, dehydrated brains underwent archival storage at −80°C (MDF-382ECN, SANYO). Fixed/dehydrated specimens were snap-frozen, coronally sliced into 20-μm sections via freezing microtome (CM1950, Leica), and preserved in 4°C sodium azide buffer (2%) pending staining. Tissue sections received 1-h blocking treatment using PBS containing 0.1% Triton X-100 and 10% goat serum. Primary antibodies (Table 1) incubation proceeded overnight at 4°C in blocking solution. Post-staining, sections underwent gradual temperature equilibration to ambient conditions. Three sequential 5-min PBS washes preceded 1-h room-temperature exposure to fluorophore-tagged secondary antibodies (Table 1). Following another PBS wash cycle (3 × 5 min), nuclear counterstaining employed Hoechst or DAPI (C0030/C0065, Solarbio, China; 10 min, room temperature). Final triple-rinsing with PBS preceded slide-mounting using antifade medium. For neuronal tracing studies, sections underwent direct mounting post-staining. Qualitative/quantitative examination occurred via confocal laser scanning microscopy (TCS SP8, Leica, Germany).

**TABLE 1:** Primary and secondary antibodies used in this study.

Primary antibody	Dilution	Source	Brand	Cat. #	Secondary antibody	Dilution	Brand	Cat. #
PANK4 (WB)	1:3,000	Rabbit	CST	12055S	Goat Anti-Rabbit IgG, HRP-linked Antibody	1:3000	CST	7074S
β-ACTIN	1:2,000	Rabbit	CST	4970S				
FDX1	1:3,000	Rabbit	Proteintech	12592-1-AP				
LIAS	1:3,000	Rabbit	Proteintech	11577-1-AP				
DLAT	1:5,000	Rabbit	Proteintech	13426-1-AP				
HSP70	1:5,000	Rabbit	Proteintech	10995-1-AP				
SLC31A1	1:200	Rabbit	Novus	NB100402SS	Goat Anti-Rabbit IgG H&L (Alexa Fluor^®^ 488)	1:200	Abcam	ab150077
PANK4 (IF)	1:200	Rabbit	ThermoFisher	PA564939	Goat Anti-Rabbit IgG H&L (Cy3^®^) preadsorbed	1:200	Abcam	ab6939
ATP7A	1:200	Mouse	Novus	NBP259376	Goat Anti-Mouse IgG H&L (Alexa Fluor^®^ 488)	1:300	Abcam	ab150113
ATP7B	1:100	Mouse	Abcam	ab240880				
GAPDH	1:10,000	Mouse	Abcam	ab8245	Horse Anti-Mouse IgG, HRP-linked Antibody	1:3000	CST	7076S
GFAP	1:500	Guinea pig	Oasis Biofarm	OB-PGP055	Goat Anti-Guinea pig IgG H&L (Alexa Fluor^®^ 647)	1:200	Abcam	ab150187

WB, western blotting, IF, Immunofluorescence.

### Primary cell cultures

Cerebral cortices from postnatal C57BL/6 mice (≈24 h old) yielded primary astrocytes via our established protocols (Wang et al., 2020). Post-meningeal removal, tissue dissociation utilized sequential trypsin digestion and mechanical trituration to generate single-cell suspensions. Centrifugation at 400 rpm (4 min) pelleted cells. For astrocytic cultures, pellets underwent dual resuspension cycles totaling 15 min before plating. Cells seeded onto 12-well plates at 4 × 104 density per well employed DMEM (11330–057, Gibco, United States) supplemented with 10% fetal bovine serum (FBS, 10099141C, Gibco, Australia) and 1% penicillin/streptomycin/gentamicin (C0223, Beyotime, China), maintained at 37°C/5% CO2. Initial medium replacement occurred after 24 h, followed by tri-daily changes. Following 8–10 days’ maturation, orbital shaking (260 rpm, 20 h, 37°C) eliminated adherent microglia and OPCs. Enriched astrocytes underwent trypsin-EDTA detachment (25300–054, Gibco, United States), then allocation into three or four treatment cohorts receiving LPS- and small interfering RNA (siRNA)-containing media (L-3129, Sigma-Aldrich, United States). According to the manufacturer’s instructions, astrocytes were transfected with 25 nM of the specified siRNA using siRNA-mate plus transfection reagent (G04026, GenePharma, China) in order to achieve siRNA-mediated gene silencing. Following siRNAs were employed: Pank4-Mus-1339 (GenePharma, China). After 48 h of transfection, the media were treated with LPS and PBS for 24 h before being collected for additional research.

### RNA-seq data analysis

Primary astrocytes were lysed using TRIzol^®^ Reagent (DP421; Tiangen Biotech, Beijing, China) for total RNA isolation, strictly following the manufacturer’s protocol. RNA integrity was assessed with an Agilent 5300 Bioanalyzer (RQN ≥ 6.5; 28S:18S ≥ 1.0), while purity and concentration were determined via ND-2000 spectrophotometry (NanoDrop Technologies). Only high-quality RNA samples meeting these criteria (OD260/280 = 1.8∼2.2, total RNA > 1μg) were used for library preparation. RNA purification, reverse transcription, and strand-specific library construction were performed by Shanghai Majorbio Bio-pharm Biotechnology Co., Ltd. (Shanghai, China). Libraries were quantified using Qubit 4.0 and sequenced on the NovaSeq X Plus platform (PE150) using NovaSeq Reagent Kit (Illumina, United States).

Preprocessing: Raw paired-end reads underwent adapter trimming and quality control using fastp (Chen et al., 2018) (default parameters). Alignment: Filtered reads were aligned to the reference genome in orientation-aware mode using HISAT2 (Kim et al., 2015). Transcript Assembly: Genome-guided transcript assembly was performed per sample using StringTie (Pertea et al., 2015). Quantification: Gene abundances were quantified via RSEM (Li and Dewey, 2011) and normalized as transcripts per million (TPM). Differential Expression: Differentially expressed genes (DEGs) were identified using DESeq2 (Love et al., 2014) (FDR < 0.05, |log2FC| ≥ 1) or DEGseq (Wang et al., 2010) (FDR < 0.001, |log2FC| ≥ 1). Genes satisfying these thresholds were deemed statistically significant.

### Sample collection

Following completion of all behavioral assessments, seven surviving mice per cohort were deeply anesthetized utilizing 4% chloral hydrate solution. Immediate euthanasia ensued via cervical transection with sterile surgical scissors. Hippocampal tissues were then rapidly dissected out from extracted brains over ice. These dissected samples underwent brief immersion in liquid nitrogen. After immersion lasting 30 min, hippocampal specimens were retrieved and preserved at minus eighty degrees Celsius awaiting subsequent molecular analyses.

### Inductively coupled plasma-mass spectrometry (ICP-MS)

Precisely weigh hippocampal tissue samples of each group ( ± 0.0001 g) into borosilicate glass digestion tubes. For specimens preserved in ethanol or potentially containing CO2, initiate low-temperature thermoregulation on a hotplate to volatilize residual solvents. Add 10 mL of a 10:1 (v/v) nitric-perchloric acid mixture. Commence controlled thermal dissolution using a graduated heating program. If digestates develop a brown-black coloration during processing, incrementally add small volumes of nitric acid until white fumes of perchloric acid evolve. Following cooling to ambient temperature and attainment of a colorless or pale-yellow solution, quantitatively dilute digests to 25 mL with deionized water. Homogenized digests were stored at 4°C pending analysis. Procedural blanks accompanied all sample batches throughout processing. Final copper quantification was performed via inductively coupled plasma-mass spectrometry (iCAP RQ, ThermoFisher Scientific, United States) operated in standard mode.

### Western blotting assay (WB)

Cytosolic and nuclear PANK4 proteins were isolated from frozen hippocampal tissue using the specified kit (NT-032, Invent Biotechnologies Inc., United States). In brief, homogenize 20–30 mg fresh tissue of each group in 250 μl Buffer A on ice using a pestle, centrifuge at 14,000 × g for 5 min, and collect the supernatant as the cytosolic fraction. Then, grind the pellet in 0.8 ml Buffer A, centrifuge at 500 × g for 2 min, discard the supernatant, wash the nuclear pellet in Buffer B, vortex, and centrifuge at 2,000 × g for 2 min. Finally, for denatured nuclear protein extraction, resuspend the nuclei in Buffer D, add protein extraction powder, grind, add Buffer A, centrifuge at 14,000 × g for 5 min, and collect the supernatant (nuclear fraction). As previously described in our article (Wang et al., 2020), all protein concentrations were quantified via BCA assay (P0012, Beyotime Biotechnology, China). Subsequently, aliquots underwent mixing with 5 × loading buffer and heat denaturation (100C°, 10 min). Denatured samples then underwent electrophoretic separation on 10% or 6% SDS-PAGE gels (1610183, Bio-Rad Laboratories, Inc., United States), followed by transfer onto PVDF membranes (ISEQ00010, 0.2μm, Millipore, United States). Post-transfer, membranes underwent blocking for 1 h at ambient temperature using 5% nonfat milk dissolved in TBS-T (TBS plus 0.1‰ Tween-20). Primary antibodies (Table 1) incubation proceeded overnight at 4°C. Following washes, membranes were exposed to fluorescently conjugated secondary antibodies (Table 1) for 1 h (room temperature). Finally, immunoreactive bands were detected employing a ChemiDoc™ Imaging System and quantified utilizing ImageJ software.

### Reverse transcription-quantitative PCR (RT-qPCR)

Total RNA isolation from mouse tissues and cultured astrocytes employed the Eastep^®^ Super Total RNA Extraction kit (LS1040, Promega, China). Subsequently, cDNA synthesis was conducted with GoScript Reverse Transcription Mix (A2801, Promega, China). Reverse transcription mixtures were assembled on ice. The RT protocol involved 25°C for 5 min, 42°C for 1 h, and 70°C for 15 min. Synthesized cDNA products underwent refrigeration at 4°C. Oligonucleotide sequences (forward/reverse primers) appear in Table 2. Quantitative PCR utilized GoTaq^®^ qPCR Master Mix (A6002, Promega, China). Amplification conditions comprised: initial denaturation (95°C, 10 min); then 40 cycles of 95°C for 15 s and 60°C for 60 s. All reactions ran in technical triplicates. Target gene expression quantification employed the 2-ΔΔCq method with GAPDH normalization.

**TABLE 2:** Sequences of target genes in this study.

Gene name (house mouse)	Sequence layout	Primer sequence (5′–3′)	Length
Gapdh	Forward	AGGTTGTCTCCTGCGACTTCA	21
	Reverse	TGGTCCAGGGTTTCTTACTCC	21
Pank4	Forward	CGTGCCATCCACACCAACTACC	23
	Reverse	TGCCAGCCAAGCGTTCTTTACC	22
Atp7a	Forward	ACCTCTCCAGAAACCTTGCG	20
	Reverse	TCCAGTGAGGGCTGAGCTAT	20
Atp7b	Forward	CGTGAGCCAAGTGTCTCTGT	20
	Reverse	GCACTGCTCTTCATCCCTGT	20
Slc31a1	Forward	ACAGTGCTGCACATCATCCA	20
	Reverse	TGCTCTGTGATGTCCACCAC	20

### Statistical analysis

All statistical procedures were conducted using GraphPad Prism 7 (GraphPad Software, Inc.). Significance thresholds required *p*-values below 0.05 universally. For evaluating primary effects and interactions between Days and experimental cohorts, two-way ANOVA supplemented by Tukey’s *post hoc* comparisons was implemented. Inter-group differences across three conditions were assessed via one-way ANOVA with subsequent Tukey’s testing. Two-group comparisons employed independent two-tailed Student’s *t*-tests. Results appear as mean ± SEM (standard error of the mean).

## Results

### Pank4-CKO (Pank4^f/f^;Gfap-Cre) ameliorates mild cognitively impaired behaviors among older mice

Several standardized behavioral tests were employed to assess cognitive function across three experimental mouse groups. The Y-Maze test—comprising both spatial novelty preference (novel arm test) and spontaneous alternation paradigms—alongside the NOR test, were utilized to evaluate short-term spatial recognition memory (Tan et al., 2021; Xiao et al., 2025; Zhang et al., 2024). Conversely, the Barnes Maze test assessed two distinct cognitive domains: hippocampus-dependent spatial learning and spatial memory retention (long-term) (Koronyo et al., 2015; Wang et al., 2020). Additionally, the OFT quantified spontaneous movement ability via the total distance (Zhang et al., 2024).

**FIGURE 1:**
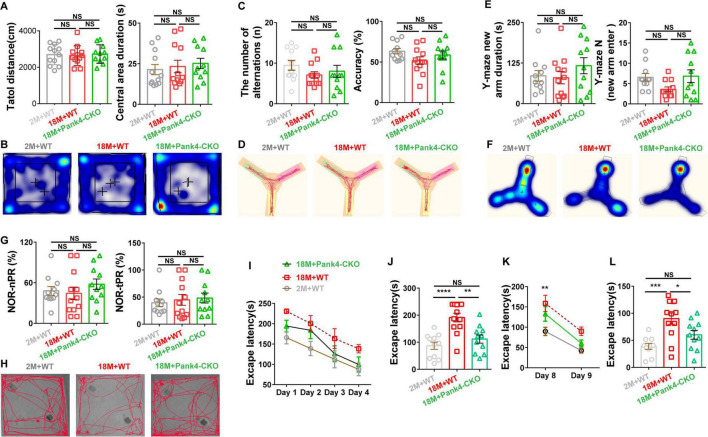
Analysis of cognitive behavior through Y-maze, NOR test, OFT, and Barnes Maze test. Aged mice at 18 months exhibited significant impairments in spatial learning and memory, as indicated by prolonged learning latency and reduced memory retention in the Barnes maze test **(I–L)**. However, no notable alterations were observed in the following behavioral parameters: spontaneous alternation rate and accuracy in the Y-maze **(C,D)**, novel arm duration and entries in the Y-maze spatial novelty preference test **(E,F)**, novel object recognition capability in the NOR test **(G,H)**, total distance traveled, or central movement duration in the OFT **(A,B)**. Importantly, early knockout of PANK4 in astrocytes (Pank4-CKO) significantly ameliorated these cognitive deficits (*n* = 11, 12, 11; One-way ANOVA, *P < 0.05, **P < 0.01, ***P < 0.001, ****P < 0.0001; 2M, two months; 18M, 18 months; WT, wild type; NOR, novel object recognition; OFT, open field test).

As shown in Figures 1A–H, no significant differences were observed among the three groups in: The number of spontaneous alternation and alternation percentage (accuracy) in the Y-maze spontaneous alternation test (Figures 1C,D); Novel arm duration and entries in the Y-maze novelty preference test (Figures 1E,F); Novel object exploration time preference ratio and duration preference ratio in the NOR test (Figures 1G,H); Total locomotion distance and center duration in the OFT (Figures 1A,B). These results indicate that aged mice exhibited no impairment in short-term spatial memory or novelty preference relative to young controls. This pattern aligns with clinical observations of cognitively normal elderly humans.

During the Barnes maze acquisition phase (days 1–4, Figure 1I), escape latency decreased significantly across all groups [Two-way ANOVA: Days Factor: *F*_(2,124)_ = 10.94, *p* < 0.0001; Groups Factor: *F*_(3,__124)_ = 15.44, *p* < 0.0001; Interaction: *F*_(6,__124)_ = 0.2142, *p* = 0.9717]. No group differences were detected on Day 4. On Day 7 (memory retention test, Figure 1J), significant intergroup differences emerged [One-way ANOVA: *F*_(2,__31)_ = 13.55, *p* < 0.0001]. *Post-hoc* analysis revealed 18-month-old wild-type mice exhibited significantly longer latencies versus 2-month-old controls (*p* < 0.0001) and aged Pank4-CKO mice (*p* = 0.0019). During reversal training (days 8–9; goal box changed each day; Figure 1K), significant group effects persisted [Two-way ANOVA: Days Factor: *F*_(2,__62)_ = 9.639, *p* = 0.0002; Groups Factor: *F*_(1,__62)_ = 33.10, *p* < 0.0001; Interaction: *F*_(2,__62)_ = 0.5083, *p* = 0.6040]. On Day 9, latency differed significantly among groups [One-way ANOVA: *F*_(2,__30)_ = 9.014, *p* = 0.0009; Figure 1L], with 18M+WT mice showing longer latencies versus young controls (*p* = 0.0006) and Pank4-CKO mice (*p* = 0.0447). Collectively, these findings indicate that astrocyte-specific Pank4 knockout attenuates age-associated deficits in long-term spatial memory retention and reversal learning. This pattern parallels the hippocampal-dependent memory impairment observed in early-stage AD (Fazio-Eynullayeva et al., 2025; Valverde et al., 2021).

### The nuclear accumulation of PANK4 from hippocampal cells in aged 18-month mice

The hippocampus serves as a critical neural substrate for memory consolidation and retrieval. Its progressive atrophy represents a well-established neuroimaging biomarker strongly correlated with MCI development (Li B. et al., 2025). This volumetric reduction likely constitutes a primary neuropathological basis for the characteristic episodic memory deficits observed in MCI populations.

**FIGURE 2:**
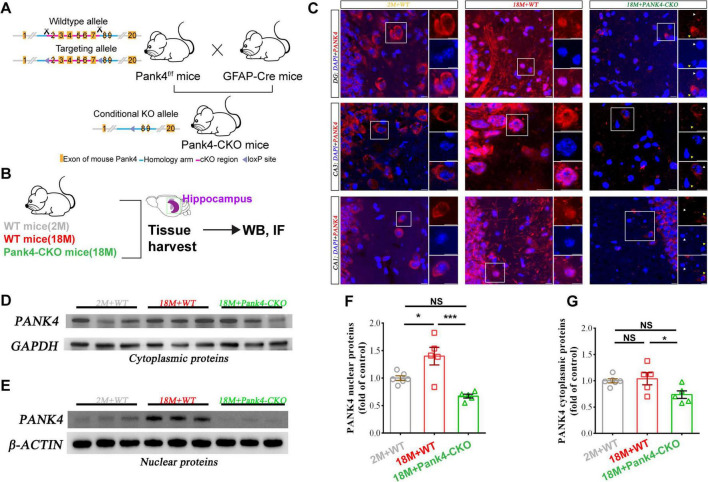
Age-induced translocation of PANK4 from cytoplasm to nucleus was assayed by immunofluorescence (IF) and Western blotting assay (WB) in mice hippocampi. Schematic of the conditional knockout strategy **(A)** and sample processing **(B)**. Under physiological conditions, PANK4 exhibits a “ring” distribution pattern, localized predominantly in the perinuclear region and cytoplasm. In contrast, 18-month-old wild-type mice demonstrated a “solid” nuclear distribution of PANK4, showing colocalization with DAPI (**C:** white arrows indicate knockout, yellow arrows denote “ring” distribution). Pank4-CKO significantly rescued this aberrant redistribution phenotype. These findings were further corroborated by nuclear **(E,F)** and cytoplasmic **(D,G)** protein fractionation assays specifically in hippocampal tissue (*n* = 5 per group for IF and *n* = 6, 5, 5 for WB; One-way ANOVA, *P < 0.05, ***P < 0.0001; 2M, two months; 18M, 18 months; WT, wild type. Scale bars = 10 μm).

Given the pivotal role of the hippocampus in spatial and temporal memory as well as learning functions, and considering that behavioral tests clearly demonstrated functional impairment in aged mice (Figure 1), we initially focused on investigating overall changes in PANK4 expression within hippocampal tissue (Figures 2A,B). In this process, we made an interesting observation: Immunofluorescence (IF) confirmed predominant cytoplasmic PANK4 staining under physiological conditions (Figure 2C and Supplementary Figure 2A), consistent with previous reports (Li et al., 2023b). In 18-month-old WT mice, we observed PANK4 nuclear accumulation characterized by translocation from cytoplasm to nucleus (Figure 2C and Supplementary Figure 2A). Interestingly, aged astrocyte-specific Pank4 knockout mice (genetically validated in Supplementary Figures 1A–D) exhibited near-complete nucleocytoplasmic redistribution of PANK4 protein (Figure 2C and Supplementary Figure 2A). As compared to the control group, it was directly seen in the aged mice’s enhanced PANK4 staining in the nucleus without corresponding cytoplasmic reduction in the CA1, CA3, and dentate gyrus (DG) pyramidal layer of the hippocampus.

To quantify age-related localization changes, we performed subcellular fractionation (Supplementary Figure 3) followed by immunoblotting as previously described (Figures 2D–G; Wang et al., 2020). Nuclear PANK4 increased significantly in 18M+WT mice versus young controls [One-way ANOVA: *F*_(2,__13)_ = 14.58, *p* = 0.0005; Figures 2E,F]. This accumulation was attenuated in Pank4-CKO mice (*p* = 0.0003 vs. 18M+WT). Cytoplasmic PANK4 levels showed an increasing trend in aged WT mice [One-way ANOVA: *F*_(2,__13)_ = 4.242, *p* = 0.0382; Figures 2D,G], with significant reduction in Pank4-CKO versus 18M+WT (*p* = 0.0488). There was no difference in total hippocampal PANK4 level between 18M+WT mice and young controls [One-way ANOVA: *F*_(2,__6)_ = 9.891, *p* = 0.0126; Supplementary Figures 2B,C]. In addition, Pank4-CKO mice showed reduced total PANK4 versus young controls (*p* = 0.0181, Supplementary Figures 2B,C), confirming successful knockout.

These biochemical findings corroborate IF observations and suggest age-dependent nuclear translocation of PANK4 in hippocampal cells may contribute to age-associated cognitive decline.

### Pank4-CKO downregulated the activation of cuproptosis in 18-month-old mice

Building on Tsvetkov et al.’s discovery of cuproptosis—a cell death pathway distinct from apoptosis, ferroptosis, pyroptosis, and necroptosis—recent evidence confirms this mechanism centers on copper-induced aggregation of lipoylated mitochondrial proteins, revealing novel intervention strategies for cognitive dysfunction (Deepika et al., 2025; Tsvetkov et al., 2022). Cuproptosis is initiated when Cu^+^ binds to lipoylated tricarboxylic acid (TCA) cycle components (e.g., dihydrolipoamide acetyltransferase, DLAT), triggering proteotoxic stress and irreversible cell death. Crucially, this process requires: FDX1 (ferredoxin 1, reduces Cu^2+^ → toxic Cu^+^); LIAS (lipoic acid synthetase, synthesizes lipoic acid for components lipoylation) (Tsvetkov et al., 2022). Given PANK4’s established role as a negative regulator of CoA biosynthesis (Dibble et al., 2022)—and CoA’s essential function for the TCA cycle—we hypothesized that PANK4 may modulate cellular susceptibility to cuproptosis.

**FIGURE 3:**
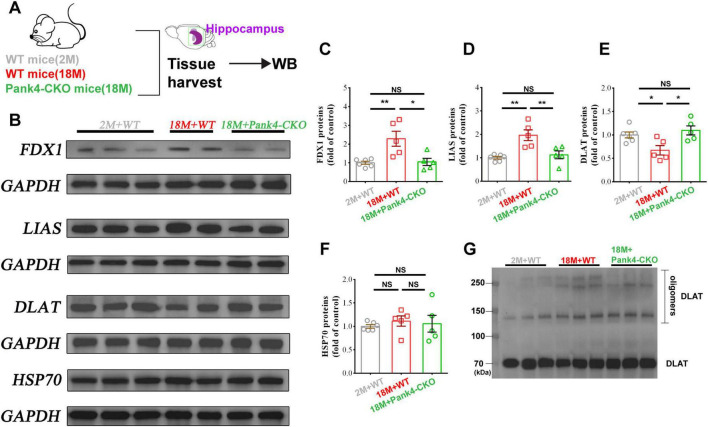
Western blotting was performed to analyze the hippocampal cuproptosis proteins. Schematic drawing depicting the sample collection of all groups **(A)**. It revealed significant upregulation of FDX1 **(B,C)** and LIAS **(B,D)**, coupled with downregulation of DLAT **(B,E)** in hippocampal tissues of 18-month-old WT mice (vs. 2M+WT). Although HSP70 **(B,F)** exhibited an upward trend, this change did not reach statistical significance. Importantly, Pank4-CKO reversed these alterations. Collectively, these findings indicate activation of the copper-depend death pathway (cuproptosis) in the hippocampus of aged mice. Changes in the level of oligomerization of DLAT **(G)** in three groups. (*n* = 6, 5, 5; One-way ANOVA, *P < 0.05, **P < 0.01; 2M, two months; 18M, 18 months; WT, wild type; FDX1, ferredoxin 1; LIAS, lipoic acid synthetase; DLAT, dihydrolipoamide S-acetyltransferase; HSP70, heat shock protein 70).

Hippocampal WB analysis (Figure 3A) revealed significant age-dependent upregulation of cuproptosis regulators in 18M+WT mice versus young controls: FDX1 increased [One-way ANOVA: *F*_(2,__13)_ = 8.739, *p* = 0.0039; Figures 3B,C]; LIAS increased [One-way ANOVA: *F*_(2,__13)_ = 10.95, *p* = 0.0016; Figures 3B,D]; DLAT showed a decreasing trend [One-way ANOVA: *F*_(2,__13)_ = 6.248, *p* = 0.01225; Figures 3B,E]. Astrocyte-specific Pank4 knockout reversed these age-associated changes: Normalized FDX1 (*p* = 0.0103 vs. 18M+WT); Normalized LIAS (*p* = 0.0075 vs. 18M+WT); Restored DLAT expression (*p* = 0.0134 vs. 18M+WT). No significant changes occurred in heat shock protein 70 (HSP70) levels [One-way ANOVA: *F*_(2,__13)_ = 0.2421, *p* = 0.7884; Figures 3B,F], indicating selective modulation of cuproptosis machinery. WB substantiated that 18M+WT mice increased the oligomerization of DLAT, an effect partially reversible with Pank4-CKO (Figure 3G). Collectively, Pank4 ablation suppresses age-induced activation of pro-cuproptotic factors (FDX1/LIAS) while restoring DLAT expression, suggesting Pank4-CKO ameliorates hippocampal cuproptosis susceptibility during aging.

### Pank4-CKO upregulated the copper transporter and reduced copper accumulation in mice hippocampi

**FIGURE 4:**
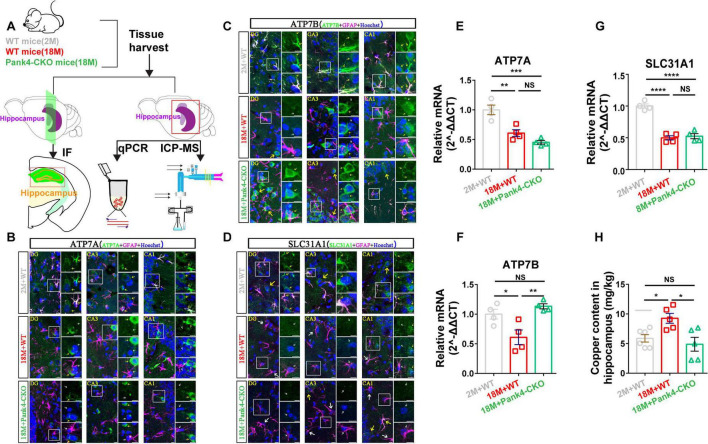
Inductively coupled plasma-mass spectrometry (ICP-MS) was used to measure the copper content, while IF and qPCR of the mouse hippocampi were used to detect copper transporters. Schematic of the all-groups experimental strategy **(A)**. IF analysis of DG, CA3, and CA1 in hippocampal tissues from 18M+WT mice revealed significant downregulation of key copper transporters: ATP7A **(B)**, ATP7B **(C)**, and SLC31A1 **(D)**, when compared to 2M+WT mice. Pank4-CKO selectively rescued the downregulation of ATP7B, but not ATP7A or SLC31A1 (white arrows: low expression; yellow arrows: high expression). This observation was further validated by qPCR analysis of hippocampal tissues **(E–G)**, confirming the protein-level changes. Treatment with Pank4-CKO clearly recovered the age-induced upregulation of copper content **(H)**, which was evidenced by the findings of the relative mRNA of copper transporters (*n* = 5 for IF, *n* = 4 for qPCR, and *n* = 6, 5, 5 for ICP-MS; One-way ANOVA, *P < 0.05, **P < 0.01, ***P < 0.001, ****P < 0.0001; 2M, two months; 18M, 18 months; WT, wild type; ATP7A, copper-transporting ATPase 1; ATP7B, copper-transporting ATPase beta; SLC31A1, solute carrier family 31 member 1; scale bars = 10 μm).

Given that cuproptosis is intrinsically linked to copper dysregulation—and considering elevated serum copper levels correlate with cognitive decline in aging (Kazemi et al., 2022)—we investigated whether PANK4 modulates hippocampal copper transporters and content. Copper homeostasis is maintained by Feng et al. (2023), Li et al. (2023a), and Wang C. et al. (2025): solute carrier family 31 member 1/copper transporter 1 (SLC31A1/CTR1): Primary copper importer; Cu-transporting ATPase 1 (ATP7A): Copper exporter (mutated in Menke’s disease) critical for neuronal copper efflux; Cu-transporting beta (ATP7B): Hepatocyte-predominant exporter (mutated in Wilson’s disease), with emerging roles in CNS copper handling. Using IF, qPCR, and ICP-MS, we quantified these transporters and copper content in hippocampal tissue from experimental groups (Figure 4A).

Hippocampal sections co-stained for copper transporters (ATP7A, ATP7B, SLC31A1) and the astrocyte marker GFAP revealed significant age-dependent changes (Figures 4B–D and Supplementary Figures 4A–C). In 18-month-old WT mice: All three transporters showed reduced immunofluorescence in CA1, CA3, and DG versus young controls (2M+WT); Astrocytic localization was confirmed by GFAP co-staining (Figures 4B–D, insets). Interestingly, astrocyte-specific Pank4 knockout: Selectively restored ATP7B expression (Figure 4C); Did not rescue ATP7A (Figure 4B) or SLC31A1 (Figure 4D). This cell-type-specific reversal indicates PANK4 regulates astrocytic ATP7B-dependent copper export during aging, providing a mechanistic basis for reduced hippocampal copper accumulation in Pank4-CKO mice.

One-way ANOVA analysis revealed significant differences between the aged and Pank4-CKO groups in the hippocampal regions in terms of the relative mRNA levels of ATP7A [*F*_(2, 9)_ = 22.29, *p* = 0.0003; Figure 4E], ATP7B [*F*_(2, 9)_ = 9.329, *p* = 0.0064; Figure 4F] and SLC31A1 [*F*_(2, 9)_ = 60.57, *p* < 0.0001; Figure 4G]. Comparing the 18-month treatment to the 2-month WT group, *post hoc* analysis showed that the transcript expression of ATP7A (*p* = 0.0031 in Figure 4E), ATP7B (*p* = 0.031 in Figure 4F), and SLC31A1 (*p* < 0.0001 in Figure 4G) was significantly reduced. While ATP7A (Figure 4E) and SLC31A1 (Figure 4G) were not saved, Pank4-CKO treatment specifically restored ATP7B (*p* = 0.0063 in Figure 4F) expression brought on by aging.

Moreover, ICP-MS was used to assess the copper content. Age and Pank4-CKO treatment had significant interaction effects on copper content, which were similar to the findings of the relative mRNA of copper transporters [One-way ANOVA: *F*_(2, 13)_ = 6.562, *p* = 0.0107; Figure 4H]. According to Tukey’s multiple comparisons, 18M+WT group showed a significant increase in the amount of copper in the mice’s hippocampi (*p* = 0.0393 in Figure 4H). In contrast, treatment with Pank4-CKO clearly recovered the age-induced upregulation of copper (*p* = 0.0115 in Figure 4H). These results were primarily consistent with earlier WB research that demonstrated once more that Pank4-CKO reduces the risk of hippocampus cuproptosis during old age.

### PANK4 mediates selective downregulation of ATP7B in LPS-stimulated astrocytes

**FIGURE 5:**
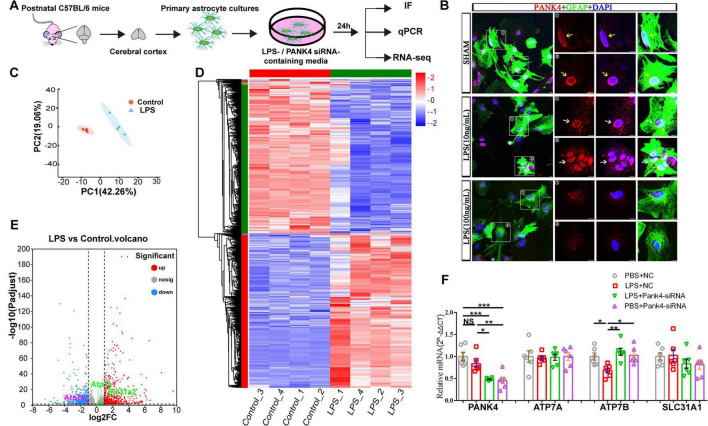
Primary astrocytes were analgised by IF, qPCR, and transcriptome RNA sequencing (RNA-seq) after LPS and PANK4 siRNA treatment. Schematic showing primary astrocytes culture strategy involving LPS or PANK4 siRNA treatment and source **(A)**. LPS (10 ng/mL) induced significant nuclear accumulation of PANK4 (**B**, white arrows: nuclear translocation; yellow arrows: “ring” distribution). In contrast, LPS (100 ng/mL) failed to elicit this redistribution **(B)**, likely due to extensive cellular toxicity. Principal component analysis (PCA) analysis of RNA-seq data **(C)** and heatmap analysis of all gene expression patterns **(D)** from astrocytes at 24 days after sham or LPS (10 ng/mL) treatment. The volcano plot analysis **(E)** showed that LPS (10 ng/mL) treatment significantly downregulated only the Atp7b gene, while expression levels of Atp7a and Slc31a2 (An important paralog of Slc31a1) remained unchanged (not significantly). Slc31a1 was not been detected. These findings were corroborated by qPCR validation at the cellular level (**F**, LPS 10 ng/mL). Treatment with PANK4 siRNA marginally increased the ATP7B transcript level but not ATP7A and SLC31A1 **(F)**. (*n* = 4 for IF and RNA-seq, *n* = 6 for qPCR; Two-way ANOVA, *P < 0.05, **P < 0.01, ***P < 0.001; Scale bars = 10 μm).

Based on the *in vivo* findings above, subsequent *in vitro* studies were conducted to further investigate the relationship between PANK4 and copper transporters (Figure 5A). Lipopolysaccharide (LPS) is well-established to induce neuroinflammation via secretion of inflammatory mediators, leading to cognitive impairment (Balakrishnan et al., 2025). To model the age-related neuroinflammatory environment associated with cognitive dysfunction in the aging brain, primary astrocytes were treated with different concentrations of LPS (10 and 100 ng/mL).

Primary astrocytes were cultured using our established protocols. Intriguingly, a significant nuclear translocation of PANK4 was observed specifically following stimulation with 10 ng/mL LPS, but not with the higher 100 ng/mL dose (Figure 5B). This dose-dependent effect is likely attributable to the cytotoxicity induced by high-dose LPS, as confirmed by TUNEL staining indicating increased cell death at 100 ng/mL (Supplementary Figure 5).

Therefore, to elucidate the impact of relevant neuroinflammation on copper transporter expression, transcriptome RNA sequencing (RNA-seq) was performed on primary astrocytes 24 h post-treatment with 10 ng/mL LPS (Figures 5C–E). Principal component analysis (PCA) revealed high within-group sample homogeneity, with quadruplicate measurements from each experimental cohort forming tight clusters in the principal component space (Figure 5C). The volcano plot analysis revealed that LPS treatment significantly downregulated only the Atp7b gene, while expression levels of Atp7a and Slc31a2 (An important paralog of Slc31a1) remained unchanged (Figure 5E). Slc31a1 was not been detected. This selective downregulation of Atp7b was subsequently validated by quantitative PCR (qPCR) in cultured cells, showing a significant decrease in ATP7B mRNA relative to controls [Two-way ANOVA: LPS Factor: *F*_(1, 20)_ = 9.073, *p* = 0.0069; Pank4-siRNA Factor: *F*_(1, 20)_ = 2.512, *p* = 0.1287; Interaction: *F*_(1, 20)_ = 7.008, *p* = 0.0155; Figure 5F]. In contrast, mRNA levels of PANK4, ATP7A, and SLC31A1 were unaffected by LPS (10 ng/mL) treatment (Figure 5F). PANK4 transcript level was decreased by approximately 3 folds in the astrocytes treated with Pank4-siRNA [Two-way ANOVA: LPS Factor: *F*_(1, 20)_ = 36.77, *p* < 0.0001; Pank4-siRNA Factor: *F*_(1, 20)_ = 0.8614, *p* = 0.3644; Interaction: *F*_(1, 20)_ = 1.574, *p* = 0.2240; Figure 5F]. Furthermore, treatment with PANK4 siRNA marginally increased the ATP7B transcript level (*p* < 0.01) but not ATP7A and SLC31A1. These findings closely mirror earlier animal studies, confirming PANK4’s essential function controlling copper balance, particularly via regulating ATP7B expression.

## Discussion

**FIGURE 6:**
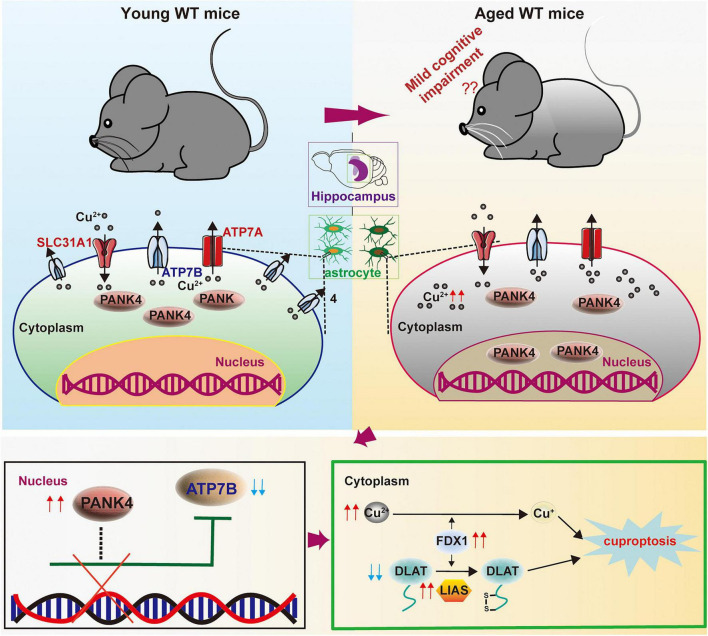
The scheme of possible mechanisms underlying the nuclear accumulation of PANK4 in hippocampal astrocytes contribution to mild cognitive impairment. WT, wild type.

MCI represents an intermediate clinical state between normative cognitive aging and dementia (Umoh, 2025). This study demonstrated (Figure 6) that nuclear accumulation of PANK4 contributes to age-associated mild cognitive decline, potentially through a mechanism involving the modulation of cuproptosis. Specifically, PANK4 regulates ATP7B-dependent copper export in astrocytes during aging. This regulatory function provides a mechanistic basis for the observed reduction in hippocampal copper accumulation in Pank4-CKO mice, which consequently attenuates cuproptosis and ameliorates cognitive deficits.

In this study, we assessed mice for signs of illness or cognitive dysfunction using the OFT, NOR test, Barnes maze, and Y-Maze (including novel arm test and spontaneous alternation tests). The OFT primarily reflects sickness-related behavioral phenotypes (Zhang et al., 2024). The NOR test evaluates object recognition memory (Tan et al., 2021). The Y-Maze quantifies short-term spatial working memory (Nishiguchi et al., 2025). Barnes maze testing, chosen as a less stressful alternative to water mazes, was employed to detect changes in spatial learning and memory (Koronyo et al., 2015). We found that 18-month-old WT mice exhibited deficits only during the long-term memory retention and reversal learning phases of the Barnes maze test. No significant differences were observed in the NOR, Y-Maze, or OFT between aged WT and the other groups. These findings indicated that the 18-month-old WT mice were in an early stage of cognitive impairment, and suggested region-specific impacts—likely confined to hippocampal circuits—rather than global brain impairment. Critically, Pank4-CKO treatment completely rescued this mild deficit (Figures 1J,L). Based on these behavioral findings, we have more precisely characterized our model as exhibiting an MCI-like phenotype centered on and characterized early by hippocampus-dependent spatial reference memory impairment. This is consistent with numerous clinical observations that spatial navigation and disorientation represent the earliest and most typical clinical manifestations in patients with MCI, especially amnestic MCI that progresses to AD (Janoutová et al., 2015; Köhler et al., 2016; Kazemi et al., 2022).

In addition, we did not find differences between young and aged mice in the OFT, Y-maze, and NOR tests, while other studies may have shown differences in these groups of mice (Barrella et al., 2026; Gao et al., 2026; Malik et al., 2023; Wang et al., 2026). Indeed, such discrepancies frequently arise not from experimental error, but rather reflect the inherent complexity of this field and the multitude of influencing factors. For example, the chronological age designated as “old” varies considerably between studies: Gao et al. chose 66 weeks+3 months+10 days (Gao et al., 2026); Barrella et al. chose 16 months (Barrella et al., 2026). Crucially, the onset and severity of age-related cognitive deficits are highly age-dependent. The specific age chosen for the “old” group in our study (75 weeks) may fall within a pre-symptomatic phase for that strain, or within a transitional phase characterized by high interindividual variability (Figure 1), and this is in line with findings from a large body of related research (Jara et al., 2026; Zhou et al., 2026). Moreover, differences in mouse strains (e.g., C57BL/6, BALB/c, DBA/2), genders, and methodological details all contribute significantly (Malik et al., 2023; Wang et al., 2026). Overall, this indicates considerable research remains necessary in this area.

Currently, no published studies report the translocation of PANK4 from the cytoplasm to the nucleus. Prior immunofluorescence analyses consistently revealed a characteristic “ring-like” distribution of PANK4 within the lens epithelium of patients aged 17, 47, and 81 years, indicating its predominant cytoplasmic localization and function (Li et al., 2023b). Significantly, we reported for the first time the nuclear accumulation of PANK4 in the hippocampi of aged mice (18-month-old) exhibiting mild cognitive impairment (Figure 2C and Supplementary Figure 2A). WB analysis confirmed that total and cytoplasmic PANK4 levels were elevated in aged mice (Figures 2D–H), consistent with previous reports of age-dependent PANK4 upregulation (Li et al., 2023b). Interestingly, Pank4 KO mice exhibited greater cataract frequency among adults compared to WT siblings (Sun et al., 2019). Collectively, these seemingly contradictory findings suggest that the cytoplasmic-to-nuclear translocation of PANK4 facilitates its nuclear accumulation, which may subsequently play an important role in progression of disease (e.g., regulate transcriptional processes).

As a pseudo-pantothenate kinase (Yao et al., 2019), PANK4 phosphorylation established role as a negative regulator of CoA biosynthesis which is an essential process governing TCA cycle flux (Dibble et al., 2022). Given the established link between mitochondrial metabolism and copper-dependent cell death (Tsvetkov et al., 2022), we investigated PANK4’s role in cuproptosis. We found that Pank4 ablation suppressed age-induced upregulation of key pro-cuproptotic factors (FDX1 and LIAS) while restoring DLAT protein expression (Figure 3). This work identifies PANK4 as a novel regulator of the cuproptosis pathway, characterized by copper-induced aggregation of lipoylated TCA cycle components. This mechanistic insight reveals potential intervention strategies for age- or other-related cognitive dysfunction (Chen et al., 2025a; Chen et al., 2024; Wang J. et al., 2025). Interestingly, while HSP70 levels showed no statistically significant change (Figure 3F), a non-significant increasing trend was observed, suggesting residual stress response activity. Future investigations should delineate cuproptosis’ precise role in MCI and evaluate its clinical relevance, potentially enabling novel diagnostic and therapeutic strategies for early-stage intervention.

While direct mechanistic links between cuproptosis and MCI remain unexplored, extant research indicates that disrupted copper homeostasis (both deficiency and excess) may indirectly contribute to MCI pathogenesis through neuronal metabolic and functional impairment (Feng et al., 2023; Ge et al., 2022). Age-dependent dysregulation is evident, with copper levels significantly increasing in the elderly (Kazemi et al., 2022)—a finding consistent with our data (Figure 4H). In AD patients’ brains, copper directly binds Aβ, promoting its aggregation and oligomerization—key processes inducing neuronal damage (Zhang et al., 2025; Zheng N. et al., 2025; Zubillaga et al., 2025). In vitro studies confirm that isolating copper from Aβ prevents accumulation, facilitates degradation, and ultimately reduces neuronal death (Behbehani et al., 2012; Kalita et al., 2022). Copper serves as an essential enzymatic cofactor yet exhibits toxicity at elevated intracellular concentrations, culminating in driving cuproptosis. Diverse mechanisms modulate copper transporters across tissues: Huang et al. demonstrated that 10-hydroxy-2-decenoic acid (10-HDA) regulates ATP7A expression, maintaining brain copper homeostasis and reducing neuronal pyroptosis post-traumatic brain injury (TBI) (Huang et al., 2025). Li et al. reported that Sorafenib upregulates TRIM21, promoting ID1 ubiquitination, increasing SLC31A1 expression, and inducing cuproptosis—a potential therapeutic strategy against ESCC (esophageal squamous cell carcinoma) (Li L. et al., 2025). Sun et al. found that ox-LDL suppresses ATP7B, causing copper overload, cuproptosis, and lipid-associated kidney injury in renal tubules (Sun et al., 2025). Significantly, we identified a cell-type-specific regulatory mechanism: PANK4 governs astrocytic ATP7B-dependent copper export during aging (Figure 4 and Supplementary Figure 4). This reversal of age-related dysregulation provided the mechanistic basis for the reduced hippocampal copper accumulation and cuproptosis observed in Pank4-CKO mice (Figure 4).

In addition, the precise nature of the interaction between PANK4 and ATP7B—whether direct or indirect—remains an open question. This uncertainty is compounded by the established complexity of ATP7B regulation, as evidenced by multiple independent mechanisms reported in the literature: (1) Mao et al. demonstrated that m6A modification of serine/arginine splicing factor 1 regulates the alternative splicing of ATP7B, thereby inhibiting copper-induced apoptosis in non-small cell lung cancer (Mao et al., 2026). (2) Epigallocatechin gallate has been shown to suppress the expression of the metal regulatory transcription factor 1 (MTF1), consequently impairing MTF1-mediated transcriptional regulation of ATP7B (Fu et al., 2025). (3) Furthermore, Wang et al. reported that Plumbagin reduces DNA methyltransferase 1 protein levels, leading to promoter hypomethylation, decreased ATP7B expression, and induction of cuproptosis in hepatocellular carcinoma (Wang C. et al., 2025). Critically, the regulatory mechanisms governing PANK4 itself are currently poorly defined. Existing reports primarily indicate a role in regulating acetyl-CoA synthesis via its phosphorylation (Dibble et al., 2022). Collectively, these findings illustrate that ATP7B is subject to complex regulation at multiple levels, including post-transcriptional splicing, transcriptional control, and epigenetic modification. Therefore, it is plausible that PANK4 could similarly influence ATP7B through one or more of these distinct regulatory axes or via a novel mechanism. To reflect this complexity and the need for further investigation into the specific nature of the PANK4-ATP7B interplay, we need more work.

Astrocytes play a pivotal role in neuronal circuits underlying learning and memory (Kim et al., 2024). Their essential functions encompass regulating copper homeostasis, providing metabolic support to neurons, and modulating synaptic transmission and plasticity (Bhattacharjee et al., 2020). Astrocytes exhibit significant copper storage capacity and resistance to copper toxicity under conditions of chronic copper exposure (Chen et al., 2008; Scheiber and Dringen, 2011). However, rapid copper influx can overwhelm these protective mechanisms (Scheiber et al., 2010). *In vitro*, excess copper decreases astrocyte viability, while subcytotoxic concentrations impair mitochondrial function, triggering reactive oxygen species (ROS) production and leading to hypertrophy or cell death (Witt et al., 2021). Our *in vitro* results (Figure 5) closely parallel the prior *in vivo* observations (Figures 2, 4), reinforcing the critical role of astrocytes in modulating copper homeostasis, specifically through its association with PANK4 which regulating ATP7B expression. In addition, Aβ activates NF-κB and complement signaling within astrocytes in AD patients. This induces the synthesis of inflammatory mediators (e.g., IL-1β, TNF-α), impairing synaptic density and dendritic morphology (Lian et al., 2016). Astrocytes also utilize mechano-gated Piezo1 channel-mediated mechanotransduction to regulate adult neurogenesis and cognitive function (Chi et al., 2022). Therefore, to investigate the interplay between astrocytic copper regulation, inflammation, and cognitive decline, we employed LPS, primary astrocyte cultures, and astrocyte-specific conditional knockout mice.

We observed that LPS stimulation successfully induced PANK4 nuclear translocation (Figure 5B). This finding further underscores the significant role of immune-inflammatory pathways in triggering age-related pathological changes. Consequently, we utilized LPS-induced PANK4 nuclear translocation as our primary *in vitro* model to subsequently validate its mechanistic impact on copper metabolism. This approach effectively addresses our core objective of utilizing cellular research to probe this specific age-related pathological mechanism. We acknowledge, however, that LPS stimulation may influence copper homeostasis through additional pathways independent of PANK4 translocation. Exploring these potential confounders and developing more targeted models will be an important focus for future research.

## Conclusion

In conclusion, our findings showed PANK4 buildup inside nuclei promotes mild cognitive deterioration during aging, likely by influencing cuproptosis pathways (Figure 6). Specifically, PANK4 controls copper efflux dependent upon ATP7B within astrocytes throughout aging, which might be one of its mechanisms. Such governance mechanistically explains diminished copper deposition found inside the hippocampus of Pank4 conditional knockout mice, thereby curbing this cell death process and lessening cognitive impairments. Of course, more research must be done in the future to support our findings. For instance, more research should be done on the possible mechanism of PANK4 translocation from CNS cells (such microglia) or other ages and brain areas (e.g., the prefrontal cortex and amygdala). It was also necessary to investigate the various performances of the female mice in this model. Here, we have offered a possible orientation for MCI research. Additionally, this work may contribute to the development of prevention and treatment techniques for mild cognitive disorders.

## Data Availability

The raw data supporting the conclusions of this article will be made available by the authors, without undue reservation.

## Footnotes

1
https://www.who.int/news-room/fact-sheets/detail/ageing-and-health

## Supplementary material

Supplementary Figure 1 IF and qPCR were used to establish that the Pank4 gene was knocked down. The distribution of PANK (red) in astrocytes (green) in the hippocampus of mice **(A,B)**. The white arrows indicated the PANK4 knockout in astrocytes **(B)**. The yellow arrows indicated the cytoplasmic PANK4 staining showed un-knockout in some other CNS cells (e.g., neurons). The negative control (without primary antibodies) indicated normal staining of GFAP and PANK4 **(C)**. The astrocyte-specific knockout of PANK4 aged (18M+Pank4-CKO) group showed lower relative mRNA levels of PANK4 compared to the control or aged WT group **(D)**, indicating that the Pank4 gene was partially knocked down (*n* = 3 for IF, *n* = 4 for qPCR; One-way ANOVA, **P* < 0.05; 2M: two months; 18M: 18 months; WT: wild type; scale bar = 10 μm).

Supplementary Figure 2 Age-induced translocation of PANK4 from cytoplasm to nucleus was assayed by immunofluorescence (IF), and total PANK4 protein was detected by WB in mice hippocampi. The white arrows indicate knockout, and yellow arrows denote “ring” distribution (**A**, low magnification). The data of total levels PANK4 in hippocampus had no difference between 18M+WT mice and young controls. Pank4-CKO mice showed reduced total PANK4 versus young controls or aged WT mice, confirming successful knockout. **(B,C)** (*n* = 3 for WB, *n* = 5 for IF; One-way ANOVA, **P* < 0.05; scale bars as indicated).

Supplementary Figure 3 Analysis of cytosolic and nuclear fractions from 18M mice hippocampus by Western blotting. Anti-Histone H3 antibody (#ab1791, Abcam, United Kingdom) was at a working dilution of 1/3,000 as well as anti-GAPDH antibody (CF, cytosolic fraction; NF, nuclear fraction).

Supplementary Figure 4 IF of the mouse hippocampi were used to detect copper transporters (low magnification). IF analysis of hippocampal tissues from 18M+WT mice revealed significant downregulation of key copper transporters: ATP7A **(A)**, ATP7B **(B)**, and SLC31A1 **(C)**, when compared to 2M+WT mice. Pank4-CKO selectively rescued the downregulation of ATP7B, but not ATP7A or SLC31A1 (white arrows: low expression; yellow arrows: high expression) (Scale bar = 50 μm, *n* = 5).

Supplementary Figure 5 After being exposed to LPS for 24 h, primary astrocytes caused apoptosis. When cells were exposed to 10 ng/mL LPS, TUNEL staining (#C1089, Beyotime Biotechnology, China) showed some apoptosis. However, following a 24-h treatment with 100 ng/mL LPS, primary astrocytes displayed a large number of TUNEL-positive cells (white arrows: TUNEL positive) (Scale bar = 50 μm, *n* = 3).

## References

[B1] Balakrishnan R. Kim Y. S. Kang S. I. Choi D. K. (2025). Green oat cognitaven^®^ attenuates mild cognitive impairment by activating the CREB/BDNF/Nrf2/HO-1 pathway and modulating NF-κB/MAPK signaling. *Biomed. Pharmacother.* 189:118295. 10.1016/j.biopha.2025.118295 40580879

[B2] Barrella L. Ramírez-Ponce M. P. Vázquez-Román V. Millán-Huang M. S. Maldonado M. D. Flores-Cordero J. A. et al. (2026). Targeting lysosomal acidification to restore microglial homeostasis and mitigate memory decline during male brain ageing. *Brain Behav. Immun.* 131:106170. 10.1016/j.bbi.2025.106170 41197685

[B3] Barritt S. A. DuBois-Coyne S. E. Dibble C. C. (2024). Coenzyme A biosynthesis: Mechanisms of regulation, function and disease. *Nat. Metab.* 6 1008–1023. 10.1038/s42255-024-01059-y 38871981

[B4] Behbehani G. R. Barzegar L. Mohebbian M. Saboury A. A. (2012). A comparative interaction between copper ions with Alzheimer’s β amyloid peptide and human serum albumin. *Bioinorg. Chem. Appl.* 2012:208641. 10.1155/2012/208641 22844264 PMC3399385

[B5] Bhattacharjee A. Ghosh S. Chatterji A. Chakraborty K. (2020). Neuron-glia: Understanding cellular copper homeostasis, its cross-talk and their contribution towards neurodegenerative diseases. *Metallomics* 12 1897–1911. 10.1039/d0mt00168f 33295934

[B6] Chen S. Zhou Y. Chen Y. Gu J. (2018). fastp: An ultra-fast all-in-one FASTQ preprocessor. *Bioinformatics* 34 i884–i890. 10.1093/bioinformatics/bty560 30423086 PMC6129281

[B7] Chen S. H. Lin J. K. Liu S. H. Liang Y. C. Lin-Shiau S. Y. (2008). Apoptosis of cultured astrocytes induced by the copper and neocuproine complex through oxidative stress and JNK activation. *Toxicol. Sci.* 102 138–149. 10.1093/toxsci/kfm292 18056745

[B8] Chen Y. Nan Y. Xu L. Dai A. Orteg R. M. M. Ma M. et al. (2025a). Polystyrene nanoplastics exposure induces cognitive impairment in mice via induction of oxidative stress and ERK/MAPK-mediated neuronal cuproptosis. *Part. Fibre Toxicol.* 22:13. 10.1186/s12989-025-00633-w 40394693 PMC12090536

[B9] Chen Y. Zhang J. Zheng W. Xu H. (2025b). Cuproptosis-related lncRNAs and genes: Potential markers for glioblastoma prognosis and treatment. *PLoS One* 20:e0315927. 10.1371/journal.pone.0315927 39913607 PMC11801720

[B10] Chen Z. Liu J. Zheng M. Mo M. Hu X. Liu C. et al. (2024). TRIM24-DTNBP1-ATP7A mediated astrocyte cuproptosis in cognition and memory dysfunction caused by Y_2_O_3_ NPs. *Sci. Total Environ.* 954:176353. 10.1016/j.scitotenv.2024.176353 39304169

[B11] Cheng Y. Wang G. Yang X. Wang Y. Li D. Zhao Y. et al. (2025). Artesunate alleviates Parkinson’s disease by targeting astrocyte MT2A to attenuate dopamine neuronal cuproptosis. *Pharmacol. Res.* 219:107895. 10.1016/j.phrs.2025.107895 40754045

[B12] Chi S. Cui Y. Wang H. Jiang J. Zhang T. Sun S. et al. (2022). Astrocytic Piezo1-mediated mechanotransduction determines adult neurogenesis and cognitive functions. *Neuron* 110 2984–2999.e8. 10.1016/j.neuron.2022.07.010 35963237

[B13] Deepika, Thakur A. Panghal A. Pundir R. Singh C. Goyal M. et al. (2025). Crosstalk between copper, Alzheimer’s disease, and melatonin. *Biometals* 38 1381–1420. 10.1007/s10534-025-00712-7 40650773

[B14] Dibble C. C. Barritt S. A. Perry G. E. Lien E. C. Geck R. C. DuBois-Coyne S. E. et al. (2022). PI3K drives the de novo synthesis of coenzyme A from vitamin B5. *Nature* 608 192–198. 10.1038/s41586-022-04984-8 35896750 PMC9352595

[B15] Fazio-Eynullayeva E. Cunnington M. Mystkowski P. Lv L. Aly A. Yee C. W. et al. (2025). Real-world healthcare resource utilization of Alzheimer’s disease in the early and advanced stages: A retrospective cohort study. *J. Med. Econ.* 28 81–88. 10.1080/13696998.2024.2442240 39668705

[B16] Feng D. Zhao Y. Li W. Li X. Wan J. Wang F. (2023). Copper neurotoxicity: Induction of cognitive dysfunction: A review. *Medicine* 102:e36375. 10.1097/MD.0000000000036375 38050287 PMC10695595

[B17] Fu Y. Hou L. Han K. Zhao C. Hu H. Yin S. (2025). Epigallocatechin gallate promotes cuproptosis via the MTF1/ATP7B axis in hepatocellular carcinoma. *Cells* 14:391. 10.3390/cells14060391 40136640 PMC11941326

[B18] Gao Y. Zhang J. Zhang X. Xing C. Xie P. Zhou Z. et al. (2026). Kai-Xin-San improves aging and associated neuroinflammation through mitochondrial autophagy. *J. Ethnopharmacol.* 355(Pt B):120721. 10.1016/j.jep.2025.120721 41067322

[B19] Ge E. J. Bush A. I. Casini A. Cobine P. A. Cross J. R. DeNicola G. M. et al. (2022). Connecting copper and cancer: From transition metal signalling to metalloplasia. *Nat. Rev. Cancer* 22 102–113. 10.1038/s41568-021-00417-2 34764459 PMC8810673

[B20] Golomb J. Kluger A. Ferris S. H. (2004). Mild cognitive impairment: Historical development and summary of research. *Dialogues Clin. Neurosci.* 6 351–367. 10.31887/DCNS.2004.6.4/jgolomb 22034453 PMC3181818

[B21] Hörtnagel K. Prokisch H. Meitinger T. (2003). An isoform of hPANK2, deficient in pantothenate kinase-associated neurodegeneration, localizes to mitochondria. *Hum. Mol. Genet.* 12 321–327. 10.1093/hmg/ddg026 12554685

[B22] Hou X. Mo X. Zhang X. Wang Q. Chen X. Lai C. et al. (2025). Cuproptosis-driven astrocyte reactivity exacerbates experimental cerebral malaria pathogenesis. *Parasit. Vectors* 18:454. 10.1186/s13071-025-07107-0 41214704 PMC12604326

[B23] Huang X. An Y. Liu J. Xu M. Li X. Chen X. et al. (2025). The neuroprotective effect of 10-hydroxy-2-decenoic acid in traumatic brain injury by inhibiting copper-mediated neuronal pyroptosis. *Phytomedicine* 142:156816. 10.1016/j.phymed.2025.156816 40318529

[B24] Janoutová J. Šerý O. Hosák L. Janout V. (2015). Is mild cognitive impairment a precursor of Alzheimer’s disease? Short review. *Cent. Eur. J. Public Health* 23 365–367. 10.21101/cejph.a4414 26841152

[B25] Jara C. Venegas-Zamora L. Park-Kang H. S. Lira M. Ricca M. Valenzuela S. et al. (2026). Early mitophagy activation by Urolithin A prevents, but late activation does not reverse, age-related cognitive impairment. *NPJ Aging* 12:54. 41786716 10.1038/s41514-026-00351-3 PMC13083899

[B26] Jia W. Zhu K. Shi J. Yong F. (2025). Association between dietary copper intake and cognitive function in American older adults: NHANES 2011-2014. *Sci. Rep.* 15:24334. 10.1038/s41598-025-09280-9 40624281 PMC12234787

[B27] Kalita S. Kalita S. Kawa A. H. Shill S. Gupta A. Kumar S. et al. (2022). Copper chelating cyclic peptidomimetic inhibits Aβ fibrillogenesis. *RSC Med. Chem.* 13 761–774. 10.1039/d2md00019a 35814930 PMC9215124

[B28] Kazemi T. Moodi M. Rajabi S. Sharifi F. Samarghandian S. Khorashadizadeh M. et al. (2022). Trace element concentration and cognitive dysfunction in elderly residents in Birjand. *Curr. Alzheimer Res.* 19 674–680. 10.2174/1567205019666220913114154 36100996

[B29] Kim D. Langmead B. Salzberg S. L. (2015). HISAT: A fast spliced aligner with low memory requirements. *Nat. Methods* 12 357–360. 10.1038/nmeth.3317 25751142 PMC4655817

[B30] Kim J. Yoo I. D. Lim J. Moon J. S. (2024). Pathological phenotypes of astrocytes in Alzheimer’s disease. *Exp. Mol. Med.* 56 95–99. 10.1038/s12276-023-01148-0 38172603 PMC10834520

[B31] Köhler C. A. Magalhaes T. F. Oliveira J. M. Alves G. S. Knochel C. Oertel-Knöchel V. et al. (2016). Neuropsychiatric disturbances in Mild Cognitive Impairment (MCI): A systematic review of population-based studies. *Curr. Alzheimer Res.* 13 1066–1082. 10.2174/1567205013666160502123129 27137220

[B32] Koronyo Y. Salumbides B. C. Sheyn J. Pelissier L. Li S. Ljubimov V. et al. (2015). Therapeutic effects of glatiramer acetate and grafted CD115^?^ monocytes in a mouse model of Alzheimer’s disease. *Brain* 138 2399–2422. 10.1093/brain/awv150 26049087 PMC4840949

[B33] Lei P. Ayton S. Bush A. I. (2021). The essential elements of Alzheimer’s disease. *J. Biol. Chem.* 296:100105. 10.1074/jbc.REV120.008207 33219130 PMC7948403

[B34] Li B. Dewey C. N. (2011). RSEM: Accurate transcript quantification from RNA-Seq data with or without a reference genome. *BMC Bioinformatics* 12:323. 10.1186/1471-2105-12-323 21816040 PMC3163565

[B35] Li B. Zeng B. Zeng P. Luo D. Yin F. Dong X. et al. (2025). Hippocampal-subfield macro- and microstructural changes in cerebral small vessel disease with mild cognitive impairment. *J. Affect. Disord.* 384 12–22. 10.1016/j.jad.2025.05.027 40339711

[B36] Li J. Q. Song J. H. Suckling J. Wang Y. J. Zuo C. T. Zhang C. et al. (2024). Disease trajectories in older adults with non-AD pathologic change and comparison with Alzheimer’s disease pathophysiology: A longitudinal study. *Neurobiol. Aging* 134 106–114. 10.1016/j.neurobiolaging.2023.11.002 38056216

[B37] Li L. Wang Y. Tian R. Yang T. Liu Y. Shan B. et al. (2025). TRIM21-mediated K11-linked ubiquitination of ID1 suppresses tumorigenesis and promotes cuproptosis in esophageal squamous cell carcinoma. *Adv. Sci.* 12:e02501. 10.1002/advs.202502501 PMC12462935 40652518

[B38] Li W. Guo Q. Li X. Huang L. Liu H. Liu S. (2025). Cuproptosis and immune microenvironment interplay in temporal lobe epilepsy: Identification of key molecular signatures and therapeutic targets. *J. Inflamm. Res.* 18 17089–17112. 10.2147/JIR.S561184 41377197 PMC12687654

[B39] Li X. Chen X. Gao X. (2023a). Copper and cuproptosis: New therapeutic approaches for Alzheimer’s disease. *Front. Aging Neurosci.* 15:1300405. 10.3389/fnagi.2023.1300405 38178962 PMC10766373

[B40] Li X. Luo L. L. Li R. F. Chen C. L. Sun M. Lin S. (2023b). Pantothenate kinase 4 governs lens epithelial fibrosis by negatively regulating pyruvate kinase M2-related glycolysis. *Aging Dis.* 14 1834–1852. 10.14336/AD.2023.0216-1 37196116 PMC10529755

[B41] Li Y. Steinberg J. Coleman Z. Wang S. Subramanian C. Li Y. et al. (2022). Proton magnetic resonance spectroscopy detects cerebral metabolic derangement in a mouse model of brain coenzyme a deficiency. *J. Transl. Med.* 20:103. 10.1186/s12967-022-03304-y 35197056 PMC8867880

[B42] Lian H. Litvinchuk A. Chiang A. C. Aithmitti N. Jankowsky J. L. Zheng H. (2016). Astrocyte-microglia cross talk through complement activation modulates amyloid pathology in mouse models of Alzheimer’s disease. *J. Neurosci.* 36 577–589. 10.1523/JNEUROSCI.2117-15.2016 26758846 PMC4710776

[B43] Lin Y. Huang K. Xu H. Qiao Z. Cai S. Wang Y. et al. (2020). Predicting the progression of mild cognitive impairment to Alzheimer’s disease by longitudinal magnetic resonance imaging-based dictionary learning. *Clin. Neurophysiol.* 131 2429–2439. 10.1016/j.clinph.2020.07.016 32829290

[B44] Liu P. Huang H. Zhou Q. Zhi Y. Wang J. Fu Y. et al. (2025). Path analysis of trace elements and physiological and biochemical indices associated to mild cognitive impairment in elderly Chinese. *Environ. Pollut.* 378:126470. 10.1016/j.envpol.2025.126470 40381678

[B45] Love M. I. Huber W. Anders S. (2014). Moderated estimation of fold change and dispersion for RNA-seq data with DESeq2. *Genome Biol.* 15:550. 25516281 10.1186/s13059-014-0550-8 PMC4302049

[B46] Malik N. Javaid S. Ashraf W. Siddique F. Rasool M. F. Alqahtani F. et al. (2023). Long-term supplementation of *Syzygium cumini* (L.) skeels concentrate alleviates age-related cognitive deficit and oxidative damage: A comparative study of young vs. old mice. *Nutrients* 15:666. 10.3390/nu15030666 36771374 PMC9921576

[B47] Mangalmurti A. Lukens J. R. (2022). How neurons die in Alzheimer’s disease: Implications for neuroinflammation. *Curr. Opin. Neurobiol.* 75:102575. 10.1016/j.conb.2022.102575 35691251 PMC9380082

[B48] Mao S. S. Wu D. Y. Cui R. H. Cheng X. Z. (2026). RBM15 mediated m6A modification of SRSF1 inhibits cuproptosis in non-small cell lung cancer by mediating ATP7B alternative splicing. *Kaohsiung J. Med. Sci.* 42:e70098. 10.1002/kjm2.70098 40923717 PMC12782256

[B49] Matos L. Gouveia A. M. Almeida H. (2015). ER stress response in human cellular models of senescence. *J. Gerontol. A Biol. Sci. Med. Sci.* 70 924–935. 10.1093/gerona/glu129 25149687

[B50] Matos L. Gouveia A. M. Almeida H. (2017). Resveratrol attenuates copper-induced senescence by improving cellular proteostasis. *Oxid. Med. Cell. Longev.* 2017:3793817. 10.1155/2017/3793817 28280523 PMC5322428

[B51] Mazhari S. Arjmand S. Eslami Shahrbabaki M. Karimi Ghoughari E. (2020). Comparing copper serum level and cognitive functioning in patients with schizophrenia and healthy controls. *Basic Clin. Neurosci.* 11 649–657. 10.32598/bcn.9.10.11.5.2116.1 33643558 PMC7878054

[B52] Mei Z. Liu J. Bennett D. A. Seyfried N. Wingo A. P. Wingo T. S. (2025). Unraveling sex differences in Alzheimer’s disease and related endophenotypes with brain proteomes. *Alzheimers Dement.* 21:e70206. 10.1002/alz.70206 40346727 PMC12064417

[B53] Miranda-Cervantes A. Fritzen A. M. Raun S. H. Hodek O. Møller L. L. V. Johann K. et al. (2025). Pantothenate kinase 4 controls skeletal muscle substrate metabolism. *Nat. Commun.* 16:345. 10.1038/s41467-024-55036-w 39746949 PMC11695632

[B54] Mo N. Tai C. Yang Y. Ling C. Zhang B. Wei L. et al. (2025). MT2A promotes angiogenesis in chronically ischemic brains through a copper-mitochondria regulatory mechanism. *J. Transl. Med.* 23:162. 10.1186/s12967-025-06163-5 39915841 PMC11800420

[B55] Nishiguchi T. Yamanishi K. Shimura A. Seki T. Ishii T. Aoyama B. et al. (2025). The age-related susceptibility to postoperative delirium quantified by bispectral electroencephalography correlates with postoperative delirium-like behavior in mice. *PCN Rep.* 4:e70142. 10.1002/pcn5.70142 40625340 PMC12229948

[B56] Overton M. Pihlsgård M. Elmståhl S. (2019). Prevalence and incidence of mild cognitive impairment across subtypes, age, and sex. *Dement. Geriatr. Cogn. Disord.* 47 219–232. 10.1159/000499763 31311017

[B57] Patel S. Thornton A. Parmar M. S. (2025). Resveratrol’s multifaceted potential in Alzheimer’s disease: Insights from preclinical and clinical evidence. *Mol. Neurobiol.* 62 16229–16260. 10.1007/s12035-025-05234-4 40748433

[B58] Pertea M. Pertea G. M. Antonescu C. M. Chang T. C. Mendell J. T. Salzberg S. L. (2015). StringTie enables improved reconstruction of a transcriptome from RNA-seq reads. *Nat. Biotechnol.* 33 290–295. 10.1038/nbt.3122 25690850 PMC4643835

[B59] Petersen R. C. Smith G. E. Ivnik R. J. Tangalos E. G. Schaid D. J. Thibodeau S. N. et al. (1995). Apolipoprotein E status as a predictor of the development of Alzheimer’s disease in memory-impaired individuals. *JAMA* 273 1274–1278. 10.1001/jama.1995.03520400044042 7646655

[B60] Qing Y. Zheng J. Tang T. Luo Y. Han D. Wang L. et al. (2025). The role of metal element exposure and oxidative stress in mild cognitive impairment: Evidence from a Shanghai elderly cohort. *Ecotoxicol. Environ. Saf.* 302:118617. 10.1016/j.ecoenv.2025.118617 40618494

[B61] Scheiber I. F. Dringen R. (2011). Copper-treatment increases the cellular GSH content and accelerates GSH export from cultured rat astrocytes. *Neurosci. Lett.* 498 42–46. 10.1016/j.neulet.2011.04.058 21571036

[B62] Scheiber I. F. Schmidt M. M. Dringen R. (2010). Zinc prevents the copper-induced damage of cultured astrocytes. *Neurochem. Int.* 57 314–322. 10.1016/j.neuint.2010.06.010 20600438

[B63] Singh K. K. Kumar M. Kumar P. Gupta M. K. Jha D. K. Kumari S. et al. (2012). “Free” copper: A new endogenous chemical mediator of inflammation in birds. *Biol Trace Elem Res.* 145, 338–48. 10.1007/s12011-011-9198-3 21938504

[B64] Squitti R. Ghidoni R. Siotto M. Ventriglia M. Benussi L. Paterlini A. et al. (2014). Value of serum nonceruloplasmin copper for prediction of mild cognitive impairment conversion to Alzheimer disease. *Ann. Neurol.* 75 574–580. 10.1002/ana.24136 24623259

[B65] Stiene-Martin A. Osborne J. G. Hauser K. F. (1991). Co-localization of proenkephalin mRNA using cRNA probes and a cell-type-specific immunocytochemical marker for intact astrocytes in vitro. *J. Neurosci. Methods* 36 119–126. 10.1016/0165-0270(91)90037-z 1712058

[B66] Sun D. Q. Zhong M. Y. Zhang J. H. Tang H. Hu B. Shen J. Q. et al. (2025). Oxidized-LDL aggravates renal injury via tubular cuproptosis. *Cell. Signal.* 132:111839. 10.1016/j.cellsig.2025.111839 40306349

[B67] Sun M. Chen C. Hou S. Li X. Wang H. Zhou J. et al. (2019). A novel mutation of PANK4 causes autosomal dominant congenital posterior cataract. *Hum. Mutat.* 40 380–391. 10.1002/humu.23696 30585370

[B68] Tan S. W. Zhao Y. Li P. Ning Y. L. Huang Z. Z. Yang N. et al. (2021). HMGB1 mediates cognitive impairment caused by the NLRP3 inflammasome in the late stage of traumatic brain injury. *J. Neuroinflammation* 18:241. 10.1186/s12974-021-02274-0 34666797 PMC8527642

[B69] Tarailis P. Lory K. Unschuld P. G. Michel C. M. Bréchet L. (2025). Self-related thought alterations associated with intrinsic brain dysfunction in mild cognitive impairment. *Sci. Rep.* 15:12279. 10.1038/s41598-025-97240-8 40210901 PMC11986127

[B70] Tsvetkov P. Coy S. Petrova B. Dreishpoon M. Verma A. Abdusamad M. et al. (2022). Copper induces cell death by targeting lipoylated TCA cycle proteins. *Science* 375 1254–1261. 10.1126/science.abf0529 35298263 PMC9273333

[B71] Umoh M. E. (2025). Social isolation, loneliness, and cognitive health. *Clin. Geriatr. Med.* 41 357–368. 10.1016/j.cger.2025.04.007 40653340 PMC12570207

[B72] Valverde A. Dunys J. Lorivel T. Debayle D. Gay A. S. Lacas-Gervais S. et al. (2021). Aminopeptidase A contributes to biochemical, anatomical and cognitive defects in Alzheimer’s disease (AD) mouse model and is increased at early stage in sporadic AD brain. *Acta Neuropathol.* 141 823–839. 10.1007/s00401-021-02308-0 33881611 PMC8113186

[B73] Wang B. Huang X. Pan X. Zhang T. Hou C. Su W. J. et al. (2020). Minocycline prevents the depressive-like behavior through inhibiting the release of HMGB1 from microglia and neurons. *Brain Behav. Immun.* 88 132–143. 10.1016/j.bbi.2020.06.019 32553784

[B74] Wang C. Wang H. Wang C. Tian T. Jin A. Liu Y. et al. (2025). Plumbagin triggers cuproptosis in hepatocellular carcinoma (HCC) via the DNA-methyltransferase 1 (DNMT1)/microRNA-302a-3p (miR-302a-3p)/ATPase copper transporting beta (ATP7B) axis. *MedComm* 6:e70312. 10.1002/mco2.70312 40761482 PMC12318818

[B75] Wang J. Zhao X. Han B. Meng K. Gao L. (2025). The up-regulation of PTBP1 expression level in patients with Insomnia by senile dementia and promote cuproptosis of nerve cell by SLC31A1. *Sleep Med.* 128 206–218. 10.1016/j.sleep.2025.01.025 39985973

[B76] Wang L. Feng Z. Wang X. Wang X. Zhang X. (2010). DEGseq: An R package for identifying differentially expressed genes from RNA-seq data. *Bioinformatics* 26 136–138. 10.1093/bioinformatics/btp612 19855105

[B77] Wang Q. Sun J. Chen T. Song S. Hou Y. Feng L. et al. (2023). Ferroptosis, pyroptosis, and cuproptosis in Alzheimer’s disease. *ACS Chem. Neurosci.* 14 3564–3587. 10.1021/acschemneuro.3c00343 37703318

[B78] Wang X. Jiang S. Di L. Li S. Chen H. Huang P. et al. (2026). Fibrinogen drives neuroinflammation and neuropathology in perioperative neurocognitive disorders. *Int. Immunopharmacol.* 168(Pt 2):115906. 10.1016/j.intimp.2025.115906 41273843

[B79] Wang Y. Li D. Xu K. Wang G. Zhang F. (2025). Copper homeostasis and neurodegenerative diseases. *Neural Regen. Res.* 20 3124–3143. 10.4103/NRR.NRR-D-24-00642 39589160 PMC11881714

[B80] Witt B. Stiboller M. Raschke S. Friese S. Ebert F. Schwerdtle T. (2021). Characterizing effects of excess copper levels in a human astrocytic cell line with focus on oxidative stress markers. *J. Trace Elem. Med. Biol.* 65:126711. 10.1016/j.jtemb.2021.126711 33486291

[B81] Wu S. Chen J. (2026). Is age-related myelinodegenerative change an initial risk factor of neurodegenerative diseases? *Neural Regen. Res.* 21 648–658. 10.4103/NRR.NRR-D-24-00848 40326982 PMC12220726

[B82] Xiang R. L. Yang Y. L. Zuo J. Xiao X. H. Chang Y. S. De Fang F. (2007). PanK4 inhibits pancreatic beta-cell apoptosis by decreasing the transcriptional level of pro-caspase-9. *Cell Res.* 17 966–968. 10.1038/cr.2007.93 17971806

[B83] Xiao B. Chu C. Lin Z. Fang T. Zhou Y. Zhang C. et al. (2025). Treadmill exercise in combination with acousto-optic and olfactory stimulation improves cognitive function in APP/PS1 mice through the brain-derived neurotrophic factor- and Cygb-associated signaling pathways. *Neural Regen. Res.* 20 2706–2726. 10.4103/NRR.NRR-D-23-01681 39105365 PMC11801291

[B84] Yao J. Subramanian C. Rock C. O. Jackowski S. (2019). Human pantothenate kinase 4 is a pseudo-pantothenate kinase. *Protein Sci.* 28 1031–1047. 10.1002/pro.3611 30927326 PMC6511746

[B85] Zhang H. Ya J. Sun M. Du X. Ren J. Qu X. (2025). Inhibition of the cGAS-STING pathway via an endogenous copper ion-responsive covalent organic framework nanozyme for Alzheimer’s disease treatment. *Chem. Sci.* 16 7215–7226. 10.1039/d4sc07963a 40144496 PMC11934151

[B86] Zhang Z. H. Wang B. Peng Y. Xu Y. W. Li C. H. Ning Y. L. et al. (2024). Identification of a hippocampus-to-zona incerta projection involved in motor learning. *Adv. Sci.* 11:e2307185. 10.1002/advs.202307185 38958448 PMC11434110

[B87] Zhao Y. Shi F. Wang L. Han Y. Pang S. Wang W. et al. (2025). Role of DLAT-mediated neuron cuproptosis in cognitive impairment induced by lead exposure. *J. Appl. Toxicol.* 46 577–589. 10.1002/jat.4908 40891127

[B88] Zheng M. Chen Z. Xie J. Yang Q. Mo M. Chen L. (2025). Yttrium oxide nanoparticles affect both cognitive and memory function by disrupting copper output in neuronal cells in a rat model. *Int. J. Nanomed.* 20 5799–5815. 10.2147/IJN.S515951 40356862 PMC12066367

[B89] Zheng N. Zhou Q. Chen Z. Xie L. Yang X. Chen Z. et al. (2025). Cuproptosis: Mechanisms and links with Alzheimer’s disease. *J. Neurophysiol.* 134 1853–1876. 10.1152/jn.00370.2025 41143863

[B90] Zhou Y. Chen Y. Zhu L. Li H. Gao Y. Xian T. et al. (2026). Fenofibrate targets PPARα-CPT1C axis to reverse aging by regulating lipid metabolism and mitochondrial function. *Pharmacol. Res.* 226:108154. 10.1016/j.phrs.2026.108154 41765248

[B91] Zizioli D. Tiso N. Guglielmi A. Saraceno C. Busolin G. Giuliani R. et al. (2016). Knock-down of pantothenate kinase 2 severely affects the development of the nervous and vascular system in zebrafish, providing new insights into PKAN disease. *Neurobiol. Dis.* 85 35–48. 10.1016/j.nbd.2015.10.010 26476142 PMC4684146

[B92] Zubillaga M. Abadin X. Ivars E. Puigròs M. Trullas R. Bellini M. J. et al. (2025). Copper overload worsens the inflammatory response of microglia to amyloid beta (Aβ) by impairing phagocytosis and promoting mitochondrial DNA-mediated NLRP3 inflammasome activation. *Redox Biol.* 87:103872. 10.1016/j.redox.2025.103872 40992081 PMC12495055

