# Supplementary figures and images for: Nuclear accumulation of PANK4 in hippocampal astrocytes aggravates cuproptosis in association with mild cognitive impairment in aged mice
Source: Front Aging Neurosci. 2026 May 13;18:1816702. doi: 10.3389/fnagi.2026.1816702 (PMC13212457; doi:10.3389/fnagi.2026.1816702)

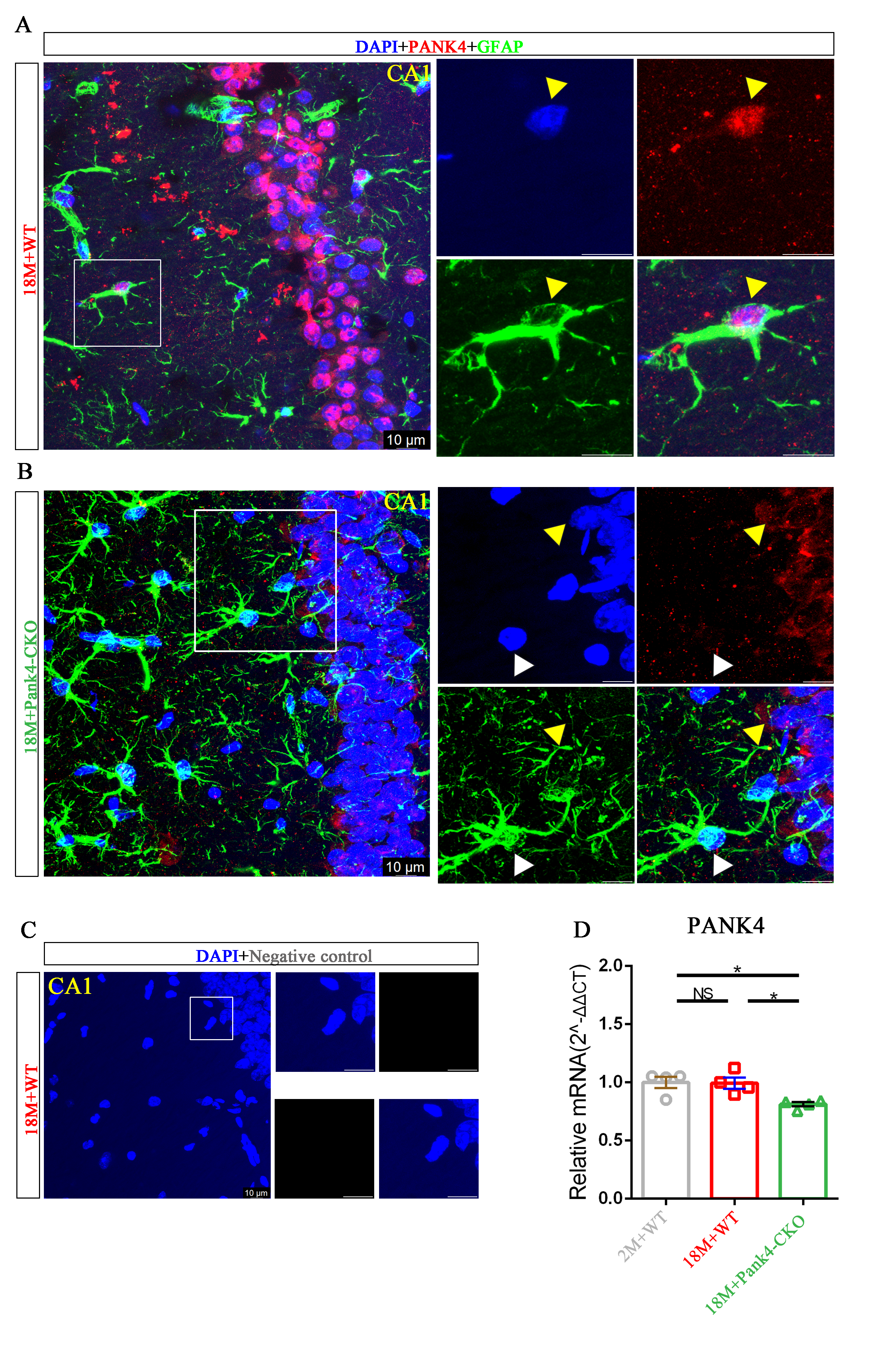

Supplement: Supplementary Figure 1 — IF and qPCR were used to establish that the Pank4 gene was knocked down. The distribution of PANK (red) in astrocytes (green) in the hippocampus of mice (A,B). The white arrows indicated the PANK4 knockout in astrocytes (B). The yellow arrows indicated the cytoplasmic PANK4 staining showed un-knockout in some other CNS cells (e.g., neurons). The negative control (without primary antibodies) indicated normal staining of GFAP and PANK4 (C). The astrocyte-specific knockout of PANK4 aged (18M+Pank4-CKO) group showed lower relative mRNA levels of PANK4 compared to the control or aged WT group (D), indicating that the Pank4 gene was partially knocked down (n = 3 for IF, n = 4 for qPCR; One-way ANOVA, *P < 0.05; 2M: two months; 18M: 18 months; WT: wild type; scale bar = 10 μm). [file Image_1.tif]

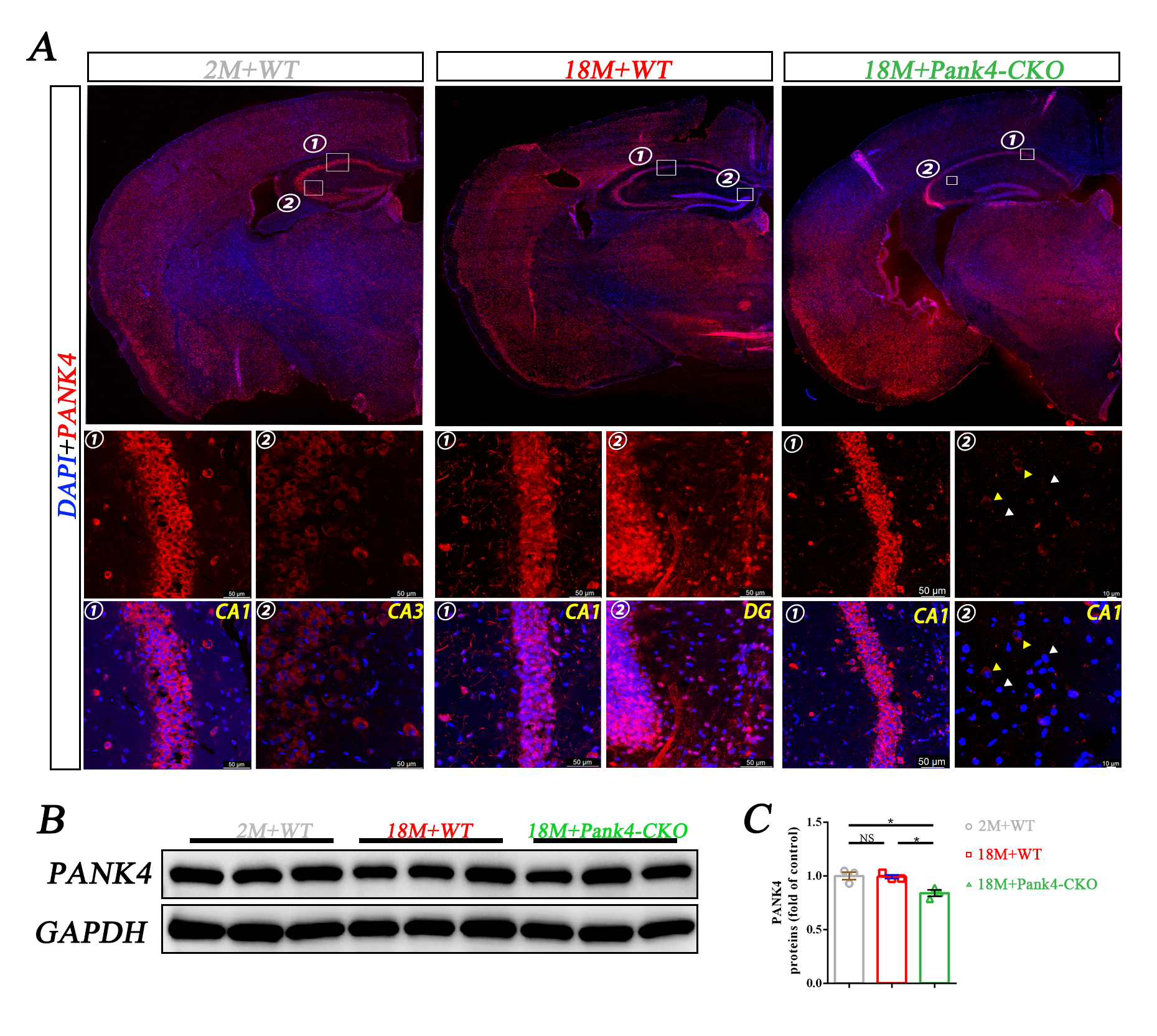

Supplement: Supplementary Figure 2 — Age-induced translocation of PANK4 from cytoplasm to nucleus was assayed by immunofluorescence (IF), and total PANK4 protein was detected by WB in mice hippocampi. The white arrows indicate knockout, and yellow arrows denote “ring” distribution (A, low magnification). The data of total levels PANK4 in hippocampus had no difference between 18M+WT mice and young controls. Pank4-CKO mice showed reduced total PANK4 versus young controls or aged WT mice, confirming successful knockout. (B,C) (n = 3 for WB, n = 5 for IF; One-way ANOVA, *P < 0.05; scale bars as indicated). [file Image_2.tif]

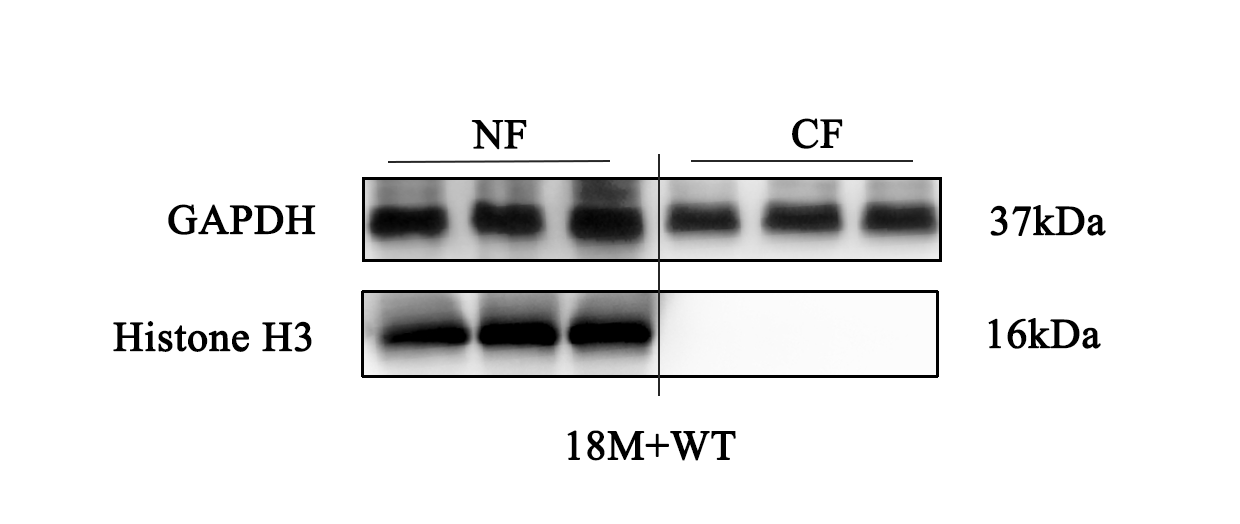

Supplement: Supplementary Figure 3 — Analysis of cytosolic and nuclear fractions from 18M mice hippocampus by Western blotting. Anti-Histone H3 antibody (#ab1791, Abcam, United Kingdom) was at a working dilution of 1/3,000 as well as anti-GAPDH antibody (CF, cytosolic fraction; NF, nuclear fraction). [file Image_3.tif]

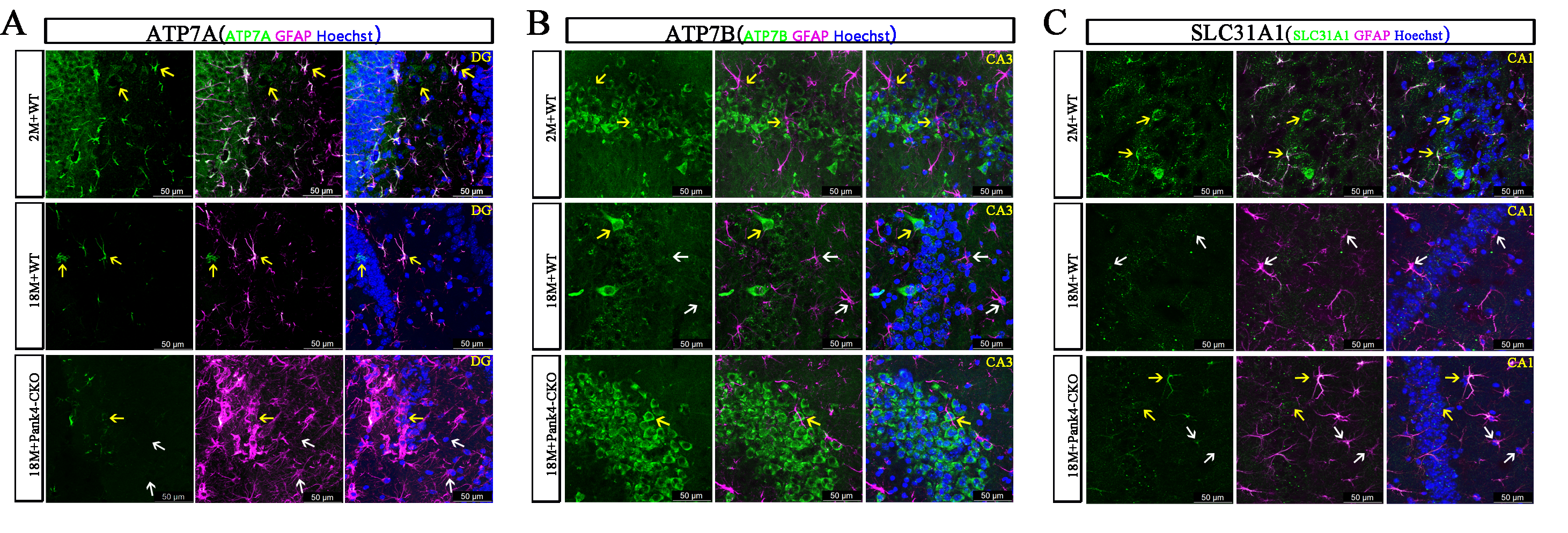

Supplement: Supplementary Figure 4 — IF of the mouse hippocampi were used to detect copper transporters (low magnification). IF analysis of hippocampal tissues from 18M+WT mice revealed significant downregulation of key copper transporters: ATP7A (A), ATP7B (B), and SLC31A1 (C), when compared to 2M+WT mice. Pank4-CKO selectively rescued the downregulation of ATP7B, but not ATP7A or SLC31A1 (white arrows: low expression; yellow arrows: high expression) (Scale bar = 50 μm, n = 5). [file Image_4.tif]

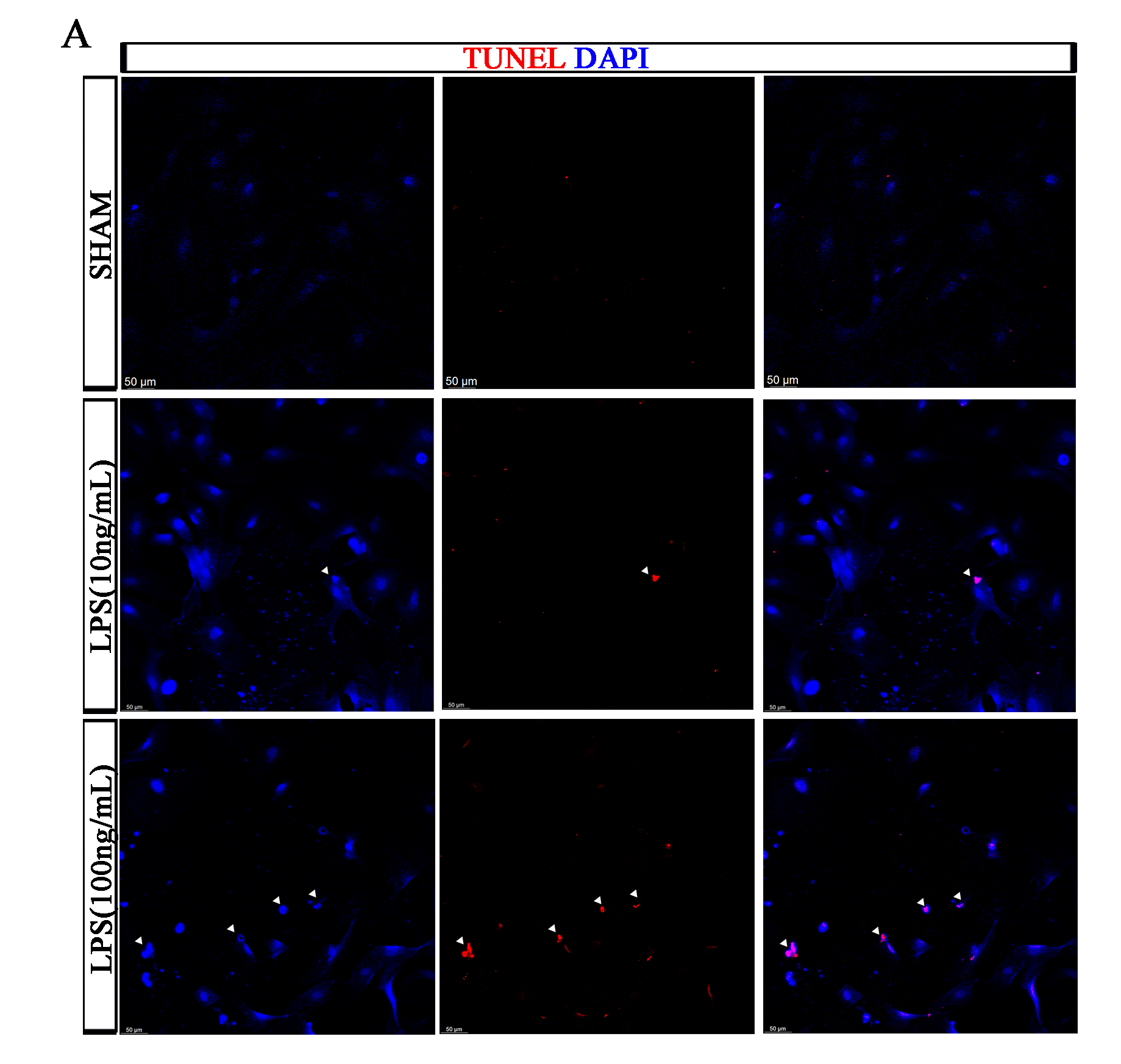

Supplement: Supplementary Figure 5 — After being exposed to LPS for 24 h, primary astrocytes caused apoptosis. When cells were exposed to 10 ng/mL LPS, TUNEL staining (#C1089, Beyotime Biotechnology, China) showed some apoptosis. However, following a 24-h treatment with 100 ng/mL LPS, primary astrocytes displayed a large number of TUNEL-positive cells (white arrows: TUNEL positive) (Scale bar = 50 μm, n = 3). [file Image_5.tif]
